# Prediction and elucidation of cellulose solubility in ionic liquids under high pressure using all-atom molecular dynamics simulations

**DOI:** 10.1039/d5ra08753h

**Published:** 2026-01-13

**Authors:** Kodai Kikuchi, Kazushi Fujimoto, Kazuyoshi Kaneko, Akio Shimizu, Tatsushi Matsuyama, Junichi Ida

**Affiliations:** a Environmental Engineering for Symbiosis, Graduate School of Science and Engineering, Soka University 1-236 Tangi Hachioji Tokyo 192-8577 Japan e24D5802@soka-u.jp ida@soka.ac.jp; b Department of Chemistry and Materials Engineering, Faculty of Chemistry, Materials and Bioengineering, Kansai University 3-3-35 Yamate-cho Suita Osaka 564-8680 Japan; c Department of Science and Engineering for Sustainable Innovation, Faculty of Science and Engineering, Soka University 1-236 Tangi Hachioji Tokyo 192-8577 Japan

## Abstract

This study investigated the pressure dependence of cellulose solubility in a 60 wt% ionic-liquid mixture of 1-ethyl-3-methylimidazolium acetate and dimethyl sulfoxide ([EMIm][OAc]/DMSO) using all-atom molecular dynamics simulations conducted over a wide pressure range (*P* = 0.1–1000 MPa) at temperature *T* = 500 K. The dissolution free energy obtained *via* the umbrella sampling method (36 windows, 200 ns each) decreased monotonically as pressure increased, indicating that solubility enhanced at elevated pressures. The underlying molecular mechanism was elucidated by performing 100 ns *NPT*-MD simulations of a 36-chain cellulose system in 60 wt% [EMIm][OAc]/DMSO at each pressure. Analysis of the radial distribution functions, coordination numbers, interaction energies, chain dispersibility, hydrogen bond populations, and conformational transitions revealed a dual mechanism: (1) pressure-induced compression strengthens solute–solvent interactions and weakens cellulose–cellulose contacts, promoting chain dispersion; and (2) conformational rearrangements of the C6 hydroxyl groups disrupt intramolecular hydrogen bonds and favor new intermolecular hydrogen bonding with [OAc]^−^ anions. This molecular-level insight demonstrated that pressure enhances cellulose solubility through both volumetric compression and active conformational mechanisms, thereby providing guidance for the design of high-pressure cellulose dissolution processes.

## Abstract 2

High pressure enhances cellulose solubility by strengthening ion–cellulose solvation through volumetric compression and by promoting new hydrogen bonding with anions *via* pressure-induced rotation of the hydroxymethyl groups.

## Graphical abstract

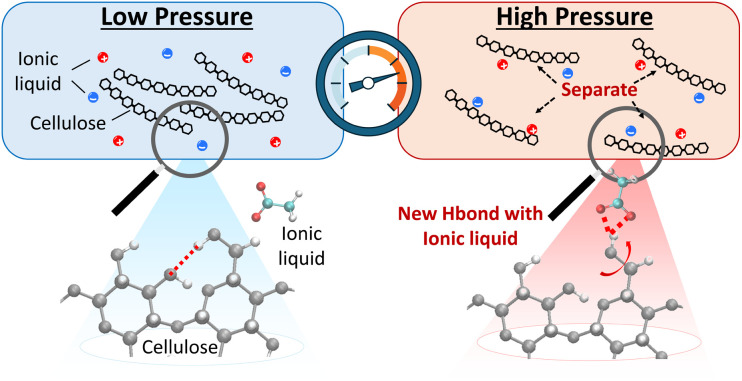

## 1 Introduction

Cellulose is the primary structural polysaccharide in plant cell walls and accounts for approximately 50% of their dry weight. It is the most abundant biomass resource on Earth and is recognized for its importance as a sustainable carbon source.^1^ Owing to its non-toxicity, biocompatibility, and biodegradability, cellulose has a diverse range of potential applications, including wound dressings,^2,3^ scaffolds for tissue engineering,^4^ and drug delivery systems.^5–7^ In environmental applications, cellulose-based materials have been explored as filtration and separation membranes,^8^ adsorbents for aqueous organic pollutants,^9,10^ and materials for heavy metal ion recovery.^11,12^ Additionally, they can be used in agriculture as water-retaining and slow-release fertilizer carriers^13–15^ and in consumer products as absorbent components in diapers and feminine hygiene products.^16^

However, natural cellulose exhibits a high degree of crystallinity (50–95%) and is stabilized by strong intra- and intermolecular hydrogen bonds, making it insoluble in common solvents like water and organic solvents.^17–20^ Therefore, solvents such as *N*-methylmorpholine-*N*-oxide (NMMO), *N*,*N*-dimethylacetamide/lithium chloride (DMAc/LiCl), dimethyl sulfoxide/tetra-*n*-butylammonium fluoride trihydrate (DMSO/TBAF), choline acetate, and aqueous sodium hydroxide/urea solutions are required. However, many of these solvents are problematic from environmental and practical standpoints because they are often highly toxic, generate volatile organic compounds, are prone to chemical degradation (limiting their reusability), and produce difficult-to-treat liquid waste or secondary pollutants.^21–23^

In recent years, ionic liquids have attracted significant attention as green solvents owing to their ability to dissolve cellulose, low volatility, low toxicity,^24^ high thermal stability, and reusability. Among the ionic liquids that can dissolve cellulose, imidazolium-based carboxylate ionic liquids and imidazolium-based halide (chloride and bromide) ionic liquids are well known. Compared to the latter, imidazolium-based carboxylate ionic liquids exhibit superior cellulose dissolution capability, lower viscosity, and lower melting points, allowing cellulose dissolution at room temperature (≈25 °C).^25^

Furthermore, Tomimatsu *et al.* demonstrated that adding DMSO as a cosolvent increased cellulose solubility, with a 60 wt% 1-alkyl-3-methylimidazolium acetate/DMSO mixture yielding the highest solubility.^26^ In this system, DMSO does not directly contribute to cellulose dissolution, since it lacks inherent cellulose-dissolving capacity, but instead reduces solution viscosity, thereby accelerating the dissolution process. Additionally, it weakens the interactions between ionic liquid cations and anions, increasing the number of free ions, which in turn enhances solvent–cellulose interactions and further increases cellulose solubility.^27^

Moreover, several studies have reported that thermal treatment increases cellulose solubility by enhancing both the dissolution rate and solubility.^28–30^ Heating is a straightforward approach from both theoretical and practical standpoints, as it requires minimal modifications to existing processes or equipment. However, thermal treatment also presents several challenges, including cellulose degradation, which may reduce mechanical strength, and caramelization reactions that cause discoloration. Additionally, heating requires relatively high energy input.

In contrast, applying pressure offers several advantages. Unlike heat, pressure generally does not break covalent bonds, requires less energy, and applies a uniform force. Therefore, understanding the effects of pressure on cellulose solubility is of considerable importance. Chang *et al.* investigated the solution structure of a cellulose/1-ethyl-3-methylimidazolium acetate ([EMIm][OAc]) system under high pressure using a diamond anvil cell at pressures of up to 3 GPa. Fourier-transform infrared spectroscopy (FT-IR) analysis revealed that pressure enhanced the interactions between cellulose and the ionic liquid, particularly between cellulose and cations.^31^ In general, stronger solute–solvent interactions are known to improve solubility. Based on this study, the application of pressure is expected to increase the solubility of cellulose in ionic liquids.

In dissolution experiments, including those involving cellulose, the dissolution process must be monitored, and the solution must be stirred. However, specialized equipment is typically required to conduct these procedures under high pressures, making such experiments technically challenging. Consequently, experimental studies on the dissolution behavior of cellulose under high-pressure conditions remain extremely limited. Chang *et al.* provided valuable insights into the interactions between cellulose and ionic liquids in high-pressure environments. However, the specific details of cellulose solubility in ionic liquids under high pressure remain largely unexplored.

Therefore, in this study, we used all-atom molecular dynamics (AA-MD) simulations to predict the solubility of cellulose in a 60 wt% [EMIm][OAc]/DMSO solvent system across a wide pressure range (*P* = 0.1–1000 MPa), which is difficult to achieve experimentally. Furthermore, we aimed to elucidate the molecular mechanisms by which pressure affects cellulose solubility.

In Section 3.1, we evaluate the validity of the force field parameters used in this study by calculating the densities of the ionic liquid, DMSO, and the ionic liquid/DMSO mixture at various temperatures and pressures through MD simulations and comparing them with experimental data. In Section 3.2, we calculate the dissolution free energy using the umbrella sampling method and use these results to predict the pressure dependence of cellulose solubility. In Section 3.3, we describe MD simulations of the cellulose/[EMIm][OAc]/DMSO system. We analyzed the radial distribution functions, coordination numbers, interaction energies, cellulose chain dispersibility, hydrogen bond populations, and conformational changes to elucidate the mechanism by which pressure affects solubility at the molecular level.

## 2 Computational methods

AA-MD simulations were conducted using the GROMACS 2023.4.^32^ The applied force field parameters included the Chemistry at HARvard Macromolecular Mechanics (CHARMM) Force Field^33,34^ for cellulose (degree of polymerization, DP = 8), Sambasivarao's parameter set^35^ for [EMIm][OAc], and AM1-BCC-derived charges^36^ for DMSO. Regarding the cellulose model, a chain with a DP of 8 was selected due to the prohibitive computational cost of simulating high-molecular-weight polymers. While this model effectively captures the essential local solute–solvent interactions governing dissolution, it represents an idealized system that excludes long-range polymer entanglement and crystalline cooperativity. Consequently, it should be noted that the calculated solubility may be overestimated compared to experimental values for high-DP cellulose. Intermolecular interactions were modeled using the Optimized Potentials for Liquid Simulations-All-Atom (OPLS-AA/L) force field,^37^ whereas intramolecular interactions were described using the General Amber Force Field (GAFF).^38^ Temperature regulation was implemented using a velocity-rescaling thermostat,^39^ and pressure control was managed with a Berendsen barostat^40^ during equilibration and a Parrinello–Rahman barostat^41,42^ for production runs. The electrostatic interactions were calculated using the Smooth Particle Mesh Ewald (SPME) method.^43^ A cutoff radius of 1.3 nm was applied to both the van der Waals interactions and the real-space component of the SPME calculation. A time step of 2 fs was considered, and all bond distances involving hydrogen atoms were constrained using a LINear Constraint Solver (LINCS) algorithm.^44^ Initial packing of the solvent molecules was performed using Packmol.^45^ Molecular and ionic structures were visualized using visual molecular dynamics (VMD).^46^

### 2.1 Force field evaluation

A cubic simulation box (6 nm × 6 nm × 6 nm) with periodic boundary conditions (PBCs) was constructed, and the molecules and ions were randomly distributed according to the compositions listed in Table 1. The system underwent energy minimization for 50 000 steps, followed by a 10 ns *NVT* (number of particles, volume, and temperature) equilibration at *T* = 500 K. Subsequently, a 10 ns *NPT* (number of particles, pressure, and temperature) equilibration and a 50 ns *NPT* production run were conducted under the temperature and pressure conditions specified in Table 1. System densities were calculated from the production trajectories, and the validity of the force field used in this study was evaluated based on the relative error with respect to the experimental density values.

**Table 1:** Calculation conditions for each system [number of pairs or molecules (*N*), temperature (*T*), and pressure (*P*)]

	[EMIm][OAc]	DMSO	60wt% [EMIm][OAc]/DMSO
Number of pairs (or molecules)	400	667	400/667
*T* (K)	293, 323, 373 (*P* = 0.1 MPa)	293, 333, 373 (*P* = 0.1 MPa)	293, 323, 373 (*P* = 0.1 MPa)
*P* (MPa)	0.1, 50, 100 (*T* = 323 K)	0.1, 15, 35 (*T* = 333 K)	0.1, 50, 100 (*T* = 323 K)

### 2.2 Umbrella sampling

#### 2.2.1 Preparation of the solvated system

The potential of mean force (PMF), which represents the free energy profile for the dissolution of a single cellulose chain from its crystal surface, was calculated using the umbrella sampling method combined with the Weighted Histogram Analysis Method (WHAM).^47,48^

**Fig. 1:**
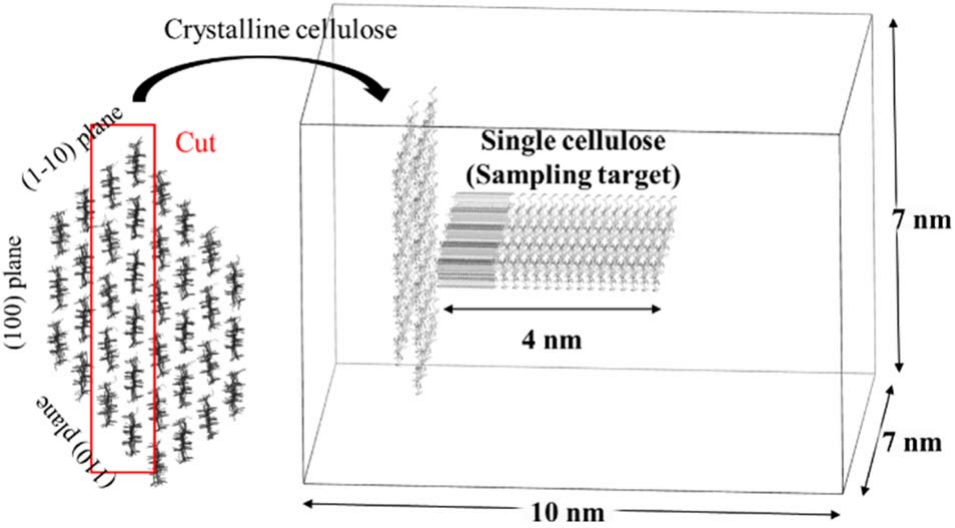
Simulation setup for umbrella sampling. The figure shows an overlay of all 36 simulation windows, representing the path of the single cellulose chain as it moved away from the crystal surface. For clarity, the solvent molecules are not shown.

The initial setup of the simulation system is illustrated in Fig. 1. An initial cellulose Iβ crystal, composed of 36 chains each with a DP of 8, was generated using cellulose-builder.^49^ The system used for the simulation was excised from this crystal and consisted of a crystal slab containing 11 cellulose chains (DP = 8) and a single isolated cellulose chain (DP = 8). These components were placed in a 7 nm × 7 nm × 10 nm simulation box with PBCs. The box was then solvated with 1150 [EMIm][OAc] ion pairs and 1670 DMSO molecules to obtain a 60 wt% [EMIm][OAc]/DMSO solvent mixture.

The reaction coordinate for the dissolution process is defined as the distance between the center-of-mass (COM) of the crystal slab and the single chain. This coordinate was chosen because it intuitively and directly describes the physical process of dissolution, specifically, the dissociation of the solute molecule from the solid surface into the bulk solvent. Sarmad *et al.*^50^ used a similar approach in recent studies.^50^ As shown in Fig. 1 and Table S1, this distance was sampled across 36 windows. The windows were spaced at 0.05 nm intervals from 0.6 to 1.55 nm (20 windows) and at 0.2 nm intervals from 1.6 to 4.6 nm (16 windows). In each window, the COM distance was constrained by the harmonic biasing potential. A strong force constant of 10 000 kJ mol^−1^ nm^−2^ was applied for the first 10 windows (0.6–1.05 nm), where strong intermolecular forces were expected. For the remaining 26 windows (1.1–4.6 nm), a weaker force constant of 1000 kJ mol^−1^ nm^−2^ was used.

All molecular dynamics simulations in this study were performed at an elevated temperature (*T* = 500 K). This relatively high temperature was chosen because the ionic liquid used as the solvent exhibits extremely high viscosity, particularly under high-pressure conditions, where both translational and conformational relaxations are significantly slowed. At ambient temperatures (*e.g.*, *T* = 298 K), the system displayed sluggish dynamics, and even submicrosecond simulations failed to yield sufficient conformational sampling for reliable statistical analysis. Under such conditions, the free energy profiles obtained from umbrella sampling exhibited poor convergence and large statistical uncertainties. Therefore, simulations were performed at an elevated temperature to accelerate molecular relaxation, enhance conformational sampling, and obtain statistically meaningful results at a feasible computational cost. The validity and possible limitations related to the elevated temperature are discussed in detail in Section 3.2.3.

#### 2.2.2 System equilibration and production run

For each window, the system was energy-minimized for 50 000 steps, followed by a 1 ns equilibration in the *NVT* ensemble at *T* = 500 K. Subsequently, a 200 ns *NPT* simulation was conducted at the same temperature (*T* = 500 K) and at each target pressure (*P* = 0.1, 200, 400, 600, 800, and 1000 MPa), where the initial 20 ns was discarded for equilibration, and the remaining 180 ns was used for production sampling. The structural integrity of the crystal slab throughout the simulation was maintained by applying a harmonic potential of 100 kJ mol^−1^ nm^−2^ to restrain the positions of the atoms.

#### 2.2.3 Analysis of the sampling data

Finally, the PMF was constructed from the biased COM distance distributions collected from all the windows using WHAM. The statistical uncertainty was estimated by dividing the last 180 ns *NPT* of the data (from 20 to 200 ns) into six independent 30 ns blocks. The final PMF profile and its error bars represent the mean and standard deviation of the results for the six blocks, respectively.

For enthalpy analysis, the average system enthalpies were calculated from the equilibrium trajectory for each of the 36 windows. The resulting plot of the enthalpy values as a function of the reaction coordinates constitutes the dissolution enthalpy profile.

### 2.3 Structural analysis of the cellulose solution (cellulose/[EMIm][OAc]/DMSO system)

The simulations for the structural analysis were performed under the same temperature and pressure conditions (*T* = 500 K; *P* = 0.1–1000 MPa) as the umbrella sampling calculations to ensure consistent sampling conditions and to elucidate the underlying mechanisms under comparable thermodynamic states.

#### 2.3.1 System preparation and simulation protocol

The structural properties of cellulose in the solution were investigated by preparing an initial system. The crystalline cellulose (DP = 8, 36 chains, Iβ-type crystal) constructed using cellulose-builder^49^ was placed at the center of a simulation box and solvated with 1500 [EMIm][OAc] ion pairs and 2180 DMSO molecules. This resulted in a system containing 10 wt% cellulose in a 60 wt% [EMIm][OAc]/DMSO solvent mixture.

A multi-step protocol was used to equilibrate the dissolved states. First, the system underwent energy minimization over 50 000 steps, followed by a 10 ns simulation in the *NVT* ensemble at *T* = 500 K to relax the solvent molecules, and then a 10 ns simulation in the *NPT* ensemble was conducted at *T* = 500 K and *P* = 0.1 MPa. Subsequently, a 400 ns *NPT* simulation was performed under the same conditions to ensure complete dissolution, which yielded a fully equilibrated cellulose solution at ambient pressure.

Using this equilibrated structure as a starting point, separate systems were prepared for higher-pressure conditions. Each system was re-equilibrated for 20 ns in the *NPT* ensemble at *T* = 500 K and the target pressures of *P* = 200, 400, 600, 800, or 1000 MPa. Finally, 100 ns *NPT* production runs were conducted for each pressure condition (including *P* = 0.1 MPa) to generate trajectories for analysis.

#### 2.3.2 Data analysis

Trajectories from the production runs were used to analyze the structural and energetic properties of the cellulose solution. Subsequently, the interaction energies, coordination numbers, radial distribution functions (RDFs), cellulose chain dispersibility, hydrogen bond populations, and cellulose dihedral angle distributions were analyzed.

**Fig. 2:**
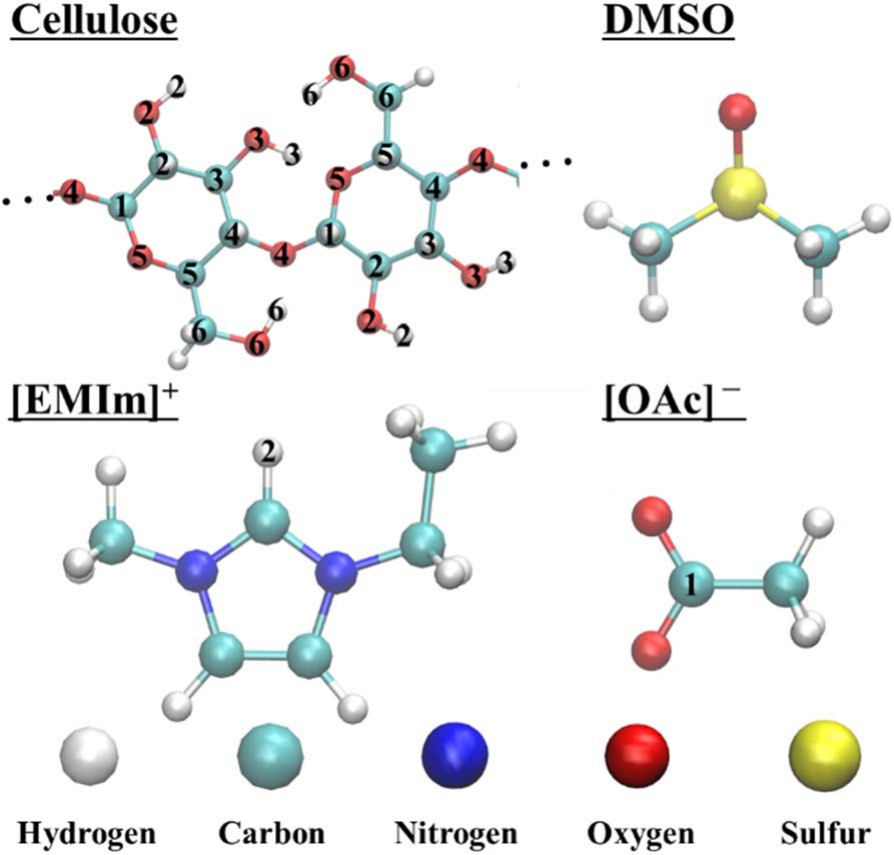
Crystalline cellulose, amorphous cellulose, cellulose chain, ionic liquid ions, and DMSO structures with their corresponding atom type labels modeled in this study.

The interaction energies, coordination numbers, and RDFs were calculated for cation–anion, cellulose–solvent (anions, cations, and DMSO), and cellulose–cellulose pairs (atom labels are shown in Fig. 2). In the cation–anion interactions, the RDF and coordination numbers were computed between the H2 and C1 atoms; in the cellulose–anion interactions, between the H atom of the OH group (H2, H3, and H6) and C1; in the cellulose–cation interactions, between the O atoms of the OH group (O2, O3, and O6) and H2; in the cellulose–DMSO interactions, between the H atom of the OH (H2, H3, and H6) and the O atoms; and in the cellulose–cellulose interactions, between the carbon atoms (C1, C2, C3, C4, C5, and C6) of the different molecules (ions).

The interaction energy (*U*_nonbonded_) between the single cellulose chain and crystalline cellulose at each pressure condition was calculated as the sum of the coulombic potential energy (*U*_Coulomb_) and van der Waals potential energy (*U*_vdW_):

1 *U*_nonbonded_ = *U*_Coulomb_ − *U*_vdW_

The cellulose distribution inhomogeneity index (*σ*_dist_) was calculated as the spatial standard deviation of the time-averaged cellulose atom counts over all the mesh cells. That is,2
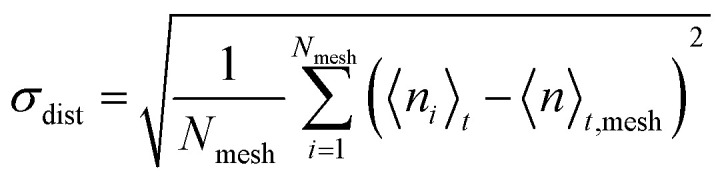
where 〈*n*_*i*_〉_*t*_ is the time-averaged number of cellulose atoms in mesh cell *i*, 〈*n*〉_*t*,mesh_ is the average of 〈*n*_*i*_〉_*t*_ over all meshes, and *N*_mesh_ = 1000 is the total number of mesh cells. A smaller *σ*_dist_ indicates a more spatially homogeneous (uniformly dispersed) cellulose distribution.

Hydrogen bonds were identified when the distance between the H atom and the acceptor atom was less than 0.35 nm, and the angle between the donor, hydrogen, and acceptor atoms exceeded 150°. In this study, donor atoms were defined as the hydroxyl O atoms in cellulose (O2, O3, and O6), whereas acceptor atoms included the hydroxyl O atoms (O2, O3, and O6), ring O atoms (O5), and glycosidic O atoms (O4) within the cellulose structure. The atom labels are presented in Fig. 2.

## 3 Results and discussion

### 3.1 Evaluation of the force field

The validity of the force field used in this study was evaluated using MD simulations to calculate the densities of [EMIm][OAc], DMSO, and 60 wt% [EMIm][OAc]/DMSO mixtures across a broad range of temperatures and pressures reported in the literature.^51–53^ The relative errors between the simulated and experimental values were calculated and are presented in Tables S2–S4. The relative errors ranged from −0.0765% to −0.9332% under all conditions, demonstrating excellent agreement with the experimental data. These results confirmed the accuracy and reliability of the force field used in this study.

### 3.2 Prediction of the pressure dependence of solubility

Kamlet–Taft (KT) parameters—the hydrogen bond acidity (*α*), basicity (*β*), and polarizability (π*)—have been widely used to describe the characteristics of solvents for cellulose dissolution. In particular, the *β* value, representing the hydrogen bond accepting ability of the solvent, is known to have a strong correlation with the cellulose solubility.^54–61^ Similarly, theoretical studies using MD simulations and density functional theory (DFT) calculations have frequently reported that the interaction energy between cellulose and the solvent, especially the strength of the hydrogen bonds, is the dominant factor governing solubility.^62–65^ These approaches, which primarily focus on the enthalpic contributions of solute–solvent interactions, are considered effective metrics for understanding the dissolution mechanism, as they show good agreement with experimental findings.

However, these discussions are centered on interaction energies and do not explicitly account for other critical thermodynamic contributions such as changes in entropy and volume that accompany dissolution. In particular, pressure is a key thermodynamic parameter that can dramatically affect the solubility by influencing the volume change (Δ*V*) and solvation structure of the system, which in turn affects the entropy (Δ*S*), as described by the fundamental relation d*G* = *V*d*P* − *S*d*T*. Therefore, a rigorous evaluation of the pressure dependence of the solubility necessitates a discussion based on the Gibbs free energy, which incorporates both enthalpic and entropic terms. In this study, we addressed this issue by using the umbrella sampling method to calculate the free energy profile along a reaction coordinate defined by pulling a single cellulose chain from its crystal. The change in this profile yielded the free energy of dissolution.

The free energy change calculated by this method, Δ*G*_dis_, corresponds to the standard Gibbs free energy of dissolution: the free energy change for the transfer of cellulose from its solid (crystalline) state to a standard state (*e.g.*, 1 mol L^−1^) in an infinitely dilute solution. The relationship between this value and the saturated solubility is given by the phase equilibrium condition, where the chemical potential of the solid is equal to that of the solute in a saturated solution:

3 Δ*G*_dis_(*P*) = −*RT* ln[*α*_sat_(*P*)] = −*RT* ln[*γ*_sat_(*P*)*X*_sat_(*P*)]

Here, *α*_sat_ is the activity of the solute (cellulose) in the saturated solution, *X*_sat_ is the solubility expressed as a mole fraction, and *γ*_sat_ is the activity coefficient at saturation. For an ideal dilute solution where solute–solute interactions are negligible (*γ*_sat_ ≈ 1), this equation would allow for the direct quantitative determination of solubility *X*_sat_ from Δ*G*_dis_.

Experimentally, the system in this study—cellulose dissolved in the [EMIm][OAc]/DMSO system at concentrations up to approximately 20 wt%—represents a concentrated polymer solution. In such systems, strong steric repulsion and intermolecular interactions among the polymer chains lead to significant nonideality. Consequently, the activity coefficient is expected to deviate substantially from unity (*γ*_sat_ ≫ 1), making a quantitative prediction of *X*_sat_ from Δ*G*_dis_ using eqn (3) extremely challenging.

Although Δ*G*_dis_(*P*) provides valuable qualitative insight into the effect of the pressure on the overall thermodynamic driving force of dissolution, it should be interpreted as an indicator of the intrinsic pressure dependence of the dissolution process rather than a direct measure of absolute solubility.

#### 3.2.1 Features of the PMF and their physical meanings

The dissolution free energy profile (*i.e.*, PMF) was first analyzed for convergence to ensure the statistical reliability of the calculations. The PMF was calculated over progressively longer sampling time ranges (20–50 ns to 20–200 ns), and the profile converged well with the 20–200 ns dataset (Fig. S1). Furthermore, sufficient overlap between the histograms of adjacent sampling windows confirmed that the statistical sampling was adequate for accurate PMF reconstruction (Fig. S2).

**Fig. 3:**
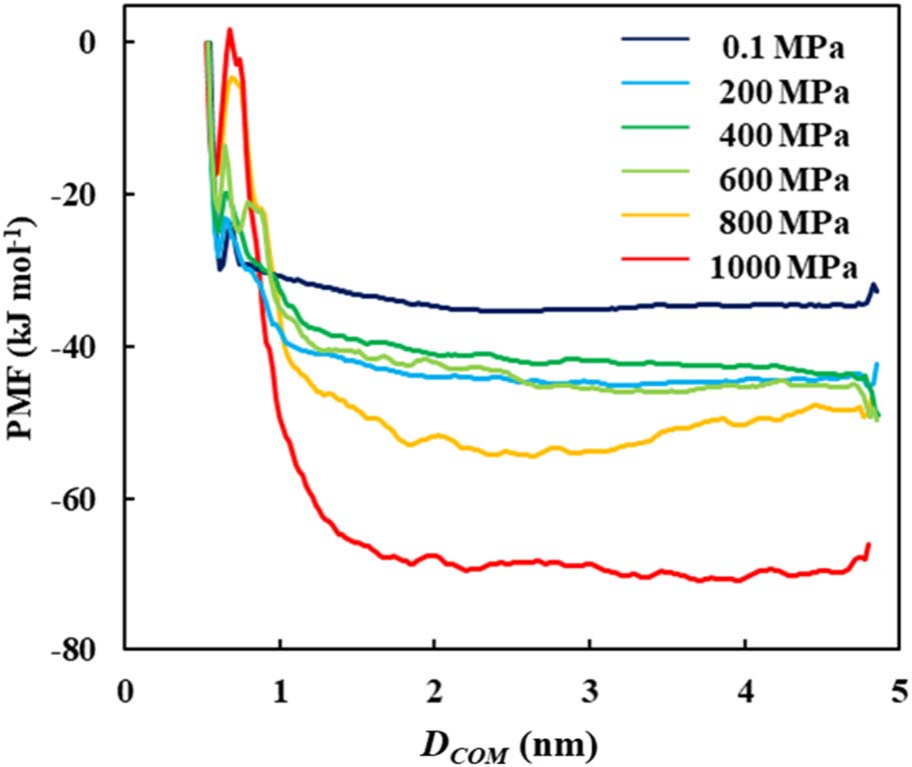
Pressure dependence of the PMF under *P* = 0.1, 200, 400, 600, 800, and 1000 MPa.

Fig. 3 illustrates the final PMF profiles as a function of the COM distance between the crystal and the single cellulose chain under each pressure condition (an enlarged view of the PMF in the range of 0.2–1.8 nm is shown in Fig. S3). All the profiles exhibited common characteristic features. A local minimum was observed at a COM distance (*D*_COM_) of approximately 0.6 nm, corresponding to the thermodynamically stable state of the single chain bound to the crystal surface. Immediately following this, a local maximum appeared at approximately *D*_COM_ = 0.7 nm, representing the activation free energy for the initial separation of the chain from the crystal. As the distance increased from *D*_COM_ = 0.7 to 2.5 nm, the PMF decreased until it converged to a stable plateau between *D*_COM_ = 2.5 and 4.5 nm. This plateau region indicates that the cellulose chain had fully dissociated from the crystal and was stably solvated by the solvent.

#### 3.2.2 Activation free energy

Before discussing the overall thermodynamics of dissolution, we analyzed the kinetic barriers. The activation free energy of dissolution (Δ*G*_act_) was calculated using the equation

4 Δ*G*_act_ = *G*^‡^ − *G*_xtal_

where *G*^‡^ is the value of the PMF at the local maximum (*D*_COM_ = 0.7 nm) and *G*_xtal_ is the value at the local minimum (*D*_COM_ = 0.6 nm).

**Fig. 4:**
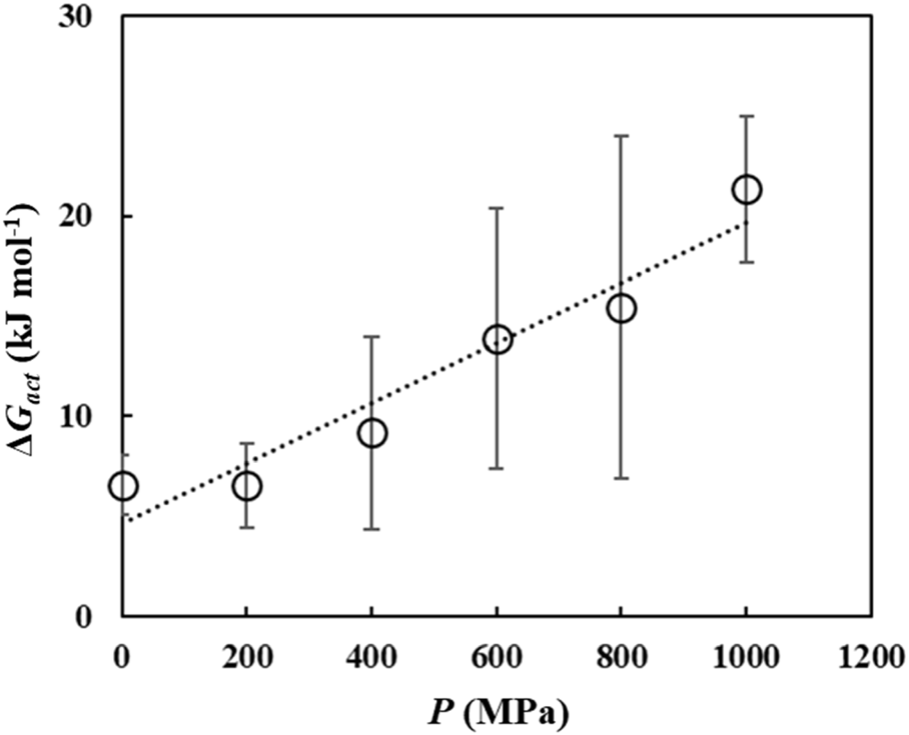
Pressure dependence of the activated free energy (Δ*G*_act_) estimated from the PMF under *P* = 0.1, 200, 400, 600, 800, and 1000 MPa.

The results show that Δ*G*_act_ increased monotonically with the applied pressure (Fig. 4 and Tables S5–S10). According to the Arrhenius-like relationship given by the equation5
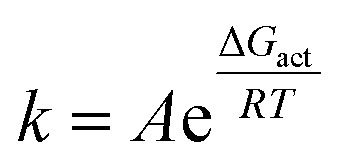
where *k* is the dissolution rate constant. The increase in the Δ*G*_act_ indicates that the dissolution rate decreases exponentially as pressure increases. Crucially, this trend is in excellent agreement with the pressure dependence of the dissolution rate that we previously reported for a similar solute–solvent system—a 36-chain cellulose crystal (DP = 8) in [EMIm][OAc]/DMSO based on direct AA-MD simulations,^66^ whereby simulations over a pressure range of *P* = 0.1–1000 MPa also revealed that the dissolution rate decreased exponentially as the pressure increased. This consistency serves as a critical validation of the approach used in this study, demonstrating that the PMF calculated using the umbrella sampling method is statistically robust and provides a reliable foundation for subsequent analysis of thermodynamic solubility.

#### 3.2.3 Prediction of the pressure dependence on solubility

The overall free energy of dissolution (Δ*G*_dis_) was determined from these profiles using the equation

6 Δ*G*_dis_ = *G*_solv_ − *G*_xtal_

where *G*_solv_ is defined as the average PMF value in the solvated plateau region (*D*_COM_ = 2.5–4.5 nm) and *G*_xtal_ is the local minimum PMF value in the bound state at 0.6 nm.

**Fig. 5:**
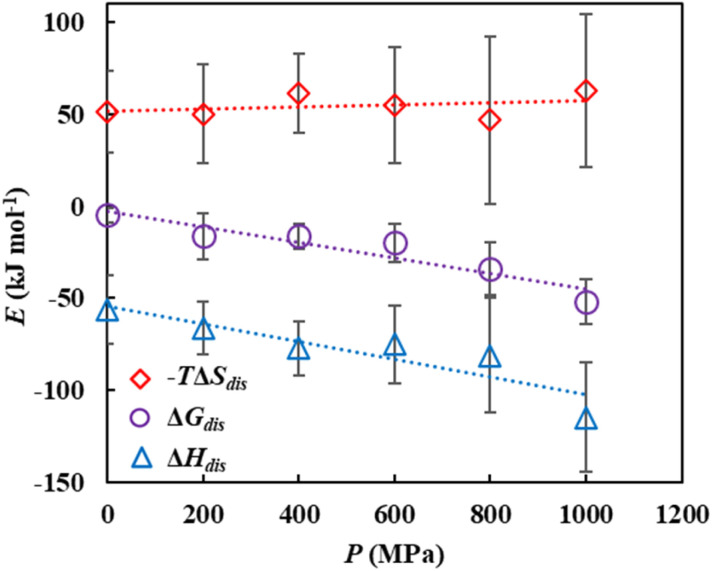
Pressure dependence of the dissolution free energy (Δ*G*_dis_), dissolution enthalpy (Δ*H*_dis_), and dissolution entropy term (−*T*Δ*S*_dis_) under *P* = 0.1, 200, 400, 600, 800, and 1000 MPa. Error bars represent the standard deviation.

The calculated values reveal a clear and consistent trend (Fig. 5 and Tables S5–S10). As the applied pressure increases, Δ*G*_dis_ decreases monotonically. This is the main finding of the present study. A lower Δ*G*_dis_ corresponds to a more spontaneous and thermodynamically favorable process, indicating that an elevated pressure acted as a strong driving force promoting the dissolution of cellulose in the [EMIm][OAc]/DMSO solvent.

However, Δ*G*_dis_ itself does not allow for the quantitative determination of solubility, as the solubility *X*_sat_(*P*) depends on the activity coefficient, which deviates significantly from unity in such non-ideal, concentrated polymer solutions. Consequently, although the observed decrease in Δ*G*_dis_ with increasing pressure clearly demonstrates that solubility is thermodynamically enhanced, it should be noted that relying solely on Δ*G*_dis_ may overestimate the extent of the actual solubility enhancement due to the large activity coefficients (*γ*_sat_ ≫ 1) in concentrated solutions. Therefore, the precise magnitude or functional form of the change in solubility cannot be inferred directly from the present calculations.

Moreover, all simulations in this study were conducted at *T* = 500 K, as discussed in Section 2.2.1. This elevated temperature was necessary to achieve sufficient conformational sampling of the cellulose and solvent molecules in a highly viscous ionic liquid medium. At lower temperatures, the molecular relaxation and diffusion of the polymer chains are extremely slow; hence, statistically converged free energy profiles cannot be obtained within feasible simulation timescales. Despite this elevated temperature, the present results capture physically reasonable dissolution behavior. For example, at ambient pressure (*P* = 0.1 MPa), the calculated dissolution free energy (Δ*G*_dis_ = −4.38 kJ mol^−1^) was negative, which is consistent with the experimentally observed solubility of cellulose in [EMIm][OAc]/DMSO mixtures.

Furthermore, numerous studies have shown that, although temperature strongly affects the absolute magnitudes of the thermophysical properties of ionic liquids, such as viscosity and density, the qualitative pressure dependence of these properties remains largely invariant with temperature.^51–53^ This indicates that the temperature primarily rescaled the dynamic timescales rather than altering the underlying pressure response. Accordingly, although the simulations were performed at an elevated temperature, the observed trend of decreasing Δ*G*_dis_ with increasing pressure is expected to be robust and physically meaningful. Nevertheless, we must acknowledge that the present findings strictly correspond to the thermodynamic conditions at *T* = 500 K and that quantitative extrapolation of the dissolution free energies to experimental temperatures should be performed with caution. Therefore, the key outcome of this study is the identification of a consistent pressure-induced enhancement of cellulose solubility, rather than the precise numerical value of Δ*G*_dis_ under ambient conditions.

### 3.3 Elucidation of the underlying mechanism

The umbrella sampling results presented in the previous section demonstrate that pressure thermodynamically promotes cellulose dissolution. In this section, we describe the mechanisms underlying this phenomenon. First, we decomposed the dissolution free energy into enthalpic and entropic contributions to identify the thermodynamic origin of the driving force (Section 3.3.1). Next, we discuss the “macroscale” changes in the solvation shells and chain dispersibility through solution structure analysis (Section 3.3.2). Finally, we investigate the specific molecular mechanisms at the “microscale”, including hydrogen bonding networks and conformational transitions, to explain the pressure-enhanced solubility in detail (Section 3.3.3).

#### 3.3.1 Decomposition of Δ*G*_dis_ into enthalpic and entropic contributions

The calculated Δ*G*_dis_ described in the preceding section was decomposed to elucidate whether the pressure-induced enhancement of solubility is driven by enthalpic (Δ*H*) or entropic (−*T*Δ*S*) contributions. The average enthalpy of the solvated state (*H*_solv_) was taken from the *D*_COM_ = 2.5–4.5 nm plateau region, whereas the enthalpy of the crystalline state (*H*_xtal_) was taken from the local minimum at approximately *D*_COM_ = 0.6 nm (Fig. 3). The enthalpy of dissolution (Δ*H*_dis_) was then calculated using the equation

7 Δ*H*_dis_ = *H*_solv_ − *H*_xtal_

The results are shown in Fig. S4. Subsequently, the entropic contribution (−*T*Δ*S*_dis_) was determined by decomposing the free energy according to the equation

8 Δ*G*_dis_ = Δ*H*_dis_ − *T*Δ*S*_dis_

Fig. 5 and Tables S11 and S12 illustrate the pressure dependence of these thermodynamic components. The entropic contribution (−*T*Δ*S*_dis_) remained largely independent of the pressure across the entire range in focus. In contrast, the enthalpic contribution (Δ*H*_dis_) decreased monotonically as the pressure increased. This clearly indicated that the pressure-induced enhancement in the cellulose solubility in the [EMIm][OAc]/DMSO system is an enthalpy-driven process.

In summary, the results presented thus far demonstrate that within *P* = 0.1–1000 MPa, increasing pressure makes the dissolution of cellulose more thermodynamically favorable, primarily due to favorable changes in the enthalpy of the system. This enthalpic origin suggests that the mechanism is rooted in the modulation of intermolecular interactions. Therefore, a detailed analysis of the interaction energies, radial distribution functions, and hydrogen bonding networks between solute–solvent and solute–solute species is necessary to elucidate the molecular-level origins of this pressure effect. These aspects will be discussed in the following sections.

#### 3.3.2 “Macroscale” analysis of the solution structure of the cellulose/[EMIm][OAc]/DMSO system

The solution structure of the dissolved cellulose was investigated at the molecular level by performing MD simulations of the cellulose/[EMIm][OAc]/DMSO system (36 cellulose chains, 1500 pairs of ionic liquids, and 2180 DMSO molecules). Visual inspection of the simulation snapshots after 100 ns confirmed that the cellulose chains remained well-dissolved and did not aggregate at any pressure (*P* = 0.1–1000 MPa) (Fig. S5).

**Fig. 6:**
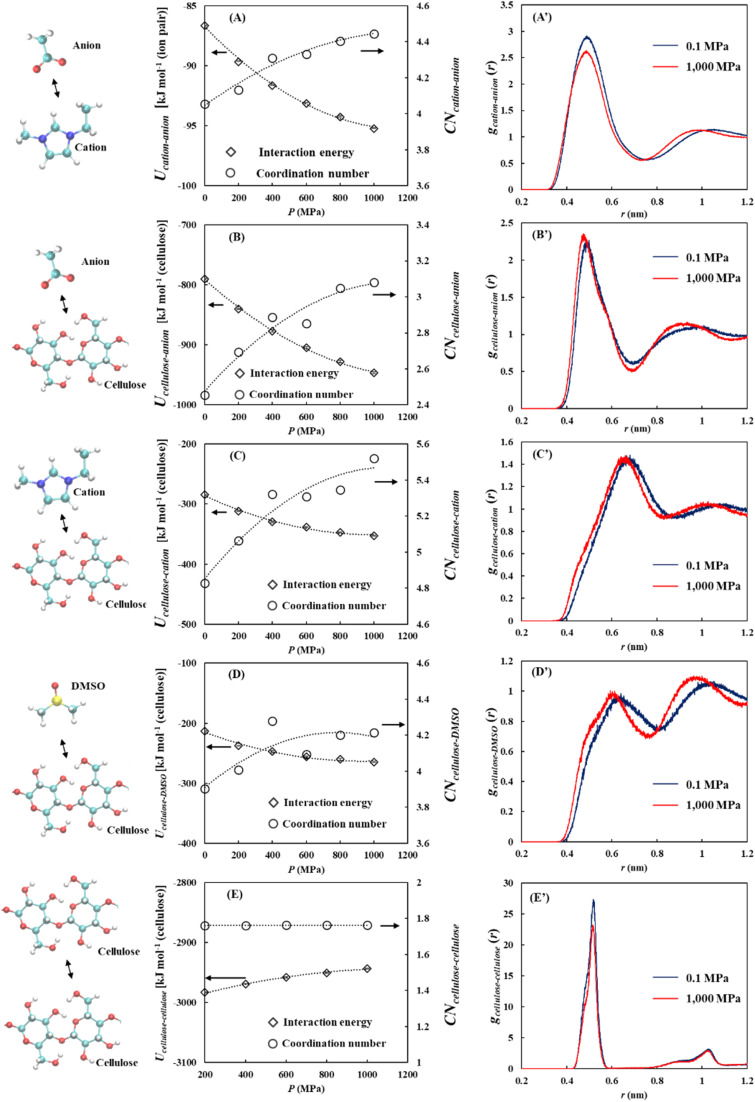
Interaction energy (*U*_nonbonded_) and coordination number (CN) and radial distribution function [*g*(*r*)] for (A and A′) cation–anion, (B and B′) cellulose–anion, (C and C′) cellulose–cation, (D and D′) cellulose–DMSO, and (E and E′) cellulose–cellulose under various pressures. Reference and target atoms for the RDF and coordination number are defined in Section 2.3.2. Error bars indicate the estimated errors.

Subsequently, the interaction energies, coordination numbers, and RDFs were calculated for the cation–anion, cellulose–solvent (anion, cation, and DMSO), and cellulose–cellulose pairs. The interaction energies and coordination numbers are summarized in Fig. 6(A–E), and the RDFs are summarized in Fig. 6(A′–E′). For clarity, the RDFs in Fig. 6(A′–E′) correspond to representative pressure conditions (*P* = 0.1 and 1000 MPa), as overlaying all pressure-dependent curves would obscure the trends. The complete set of RDFs for all pressures is shown in Fig. S6.

##### 3.3.2.1 Cation–anion interactions

As pressure increased, the cation–anion interactions became more favorable, strengthening by a factor of 1.10 from *P* = 0.1 to 1000 MPa [Fig. 6(A) and Table S13]. The anion coordination number around the cations also increased by the same factor [Fig. 6(A) and Table S14]. The RDF analysis revealed that the first and second solvation shell peaks shifted to shorter distances at higher pressures [Fig. 6(A′)]. These results indicate that pressure enhances cation–anion interactions, primarily by decreasing the interionic distance and increasing the coordination number.

##### 3.3.2.2 Cellulose–anion interactions

The cellulose–anion interactions were significantly strengthened under pressure, becoming 1.20 times more favorable at *P* = 1000 MPa than at *P* = 0.1 MPa [Fig. 6(B) and Table S15]. This was accompanied by a 1.26-fold increase in the anion coordination number around cellulose [Fig. 6(B) and Table S16]. The RDFs exhibited a corresponding shift in the solvation peaks to shorter distances [Fig. 6(B′)]. This demonstrates that pressure strengthens cellulose–anion interactions through both closer contact and increased coordination.

##### 3.3.2.3 Cellulose–cation interactions

Similarly, the cellulose–cation interactions were enhanced by a factor of 1.24 at *P* = 1000 MPa, with a 1.14-fold increase in the anion coordination number [Fig. 6(C), Tables S17 and S18]. The RDFs showed a peak shift to shorter distances and the formation of a new shoulder peak at approximately 0.4–0.5 nm at higher pressures [Fig. 6(C′)]. This shoulder suggests that the pressure induced the formation of a new, distinct solvation structure by pushing cations closer to the cellulose chain. This finding provides a basis for a molecular-level explanation of the experimental results reported by Chang *et al.*, who observed pressure-enhanced cellulose–cation interactions using FT-IR spectroscopy.^31^ Our results reveal that this enhancement is driven by a combination of shorter intermolecular distances and the formation of new solvation shells.

##### 3.3.2.4 Cellulose–DMSO interactions

The cellulose–DMSO interactions were also moderately enhanced, with the interaction energy becoming 1.20 times more favorable and the anion coordination number increasing by a factor of 1.08 at *P* = 1000 MPa [Fig. 6(D), Tables S19 and S20]. The RDFs confirmed that the cellulose–DMSO distance decreased with increasing pressure [Fig. 6(D′)].

##### 3.3.2.5 Cellulose–cellulose interactions: a counter-intuitive finding

Regarding the cellulose–cellulose interactions, the RDFs and coordination numbers [Fig. 6(E′), (E) and Table S21] indicate that the interaction strength at *P* = 1000 MPa decreases by approximately 2% compared with that at *P* = 0.1 MPa (Fig. 6(E) and Table S22). This reduction in solute–solute association may facilitate the dispersion of cellulose chains in the solvent, and such enhanced dispersion could contribute to the thermodynamic promotion of cellulose solubility under high-pressure conditions.

##### 3.3.2.6 Spatial distribution homogeneity of cellulose chains in cellulose solution

As previously discussed for the solute–solvent and solvent–solvent pairs in Sub-sections (a)–(d), increasing the pressure consistently enhanced the attractive interactions. This is consistent with the physical intuition that compression reduces the intermolecular distances [the system density of the cellulose solution increased (Fig. S7 and Table S23)]. In contrast, the cellulose–cellulose interactions discussed in Sub-section (e) exhibited a counterintuitive trend, and the interactions weakened even though the interatomic distance showed no significant change, as shown by the RDFs [Fig. 6(E′)].

**Fig. 7:**
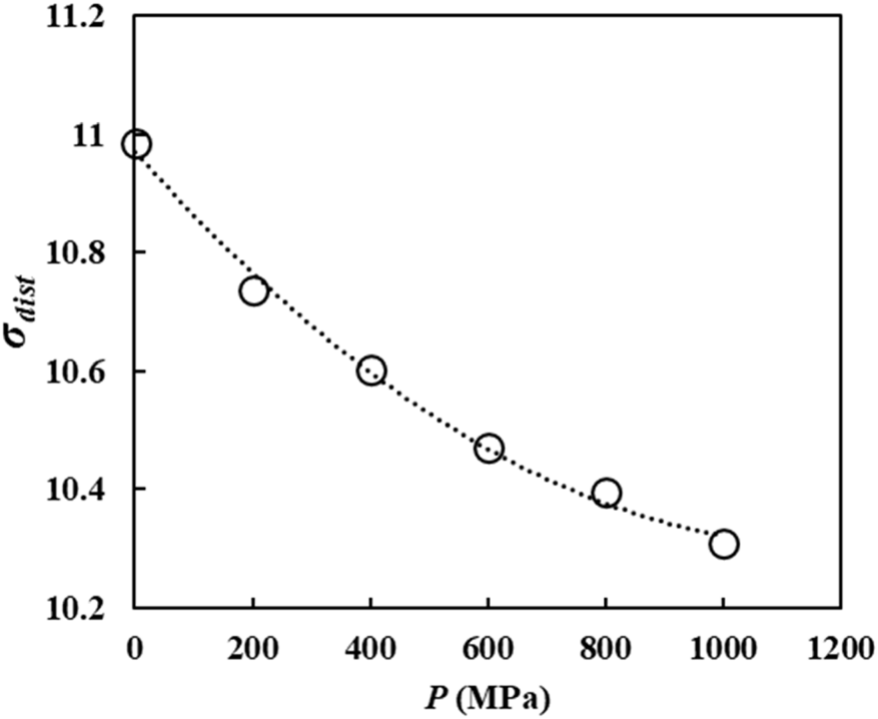
Pressure dependence of the cellulose distribution inhomogeneity index (*σ*_dist_).

We also analyzed the spatial distribution homogeneity (dispersibility) of cellulose chains in the solvent system at each pressure level to investigate the origin of this behavior. We defined a distribution inhomogeneity index (*σ*_dist_), as described in Section 2.3.2. Fig. 7 shows a plot of *σ*_dist_ as a function of pressure. This shows that *σ*_dist_ decreases as the pressure increases, confirming that cellulose becomes more homogeneously dispersed throughout the solution as the pressure increases.

##### 3.3.2.7 Summary and proposed mechanism for pressure-enhanced solubility at “macroscale”

**Fig. 8:**
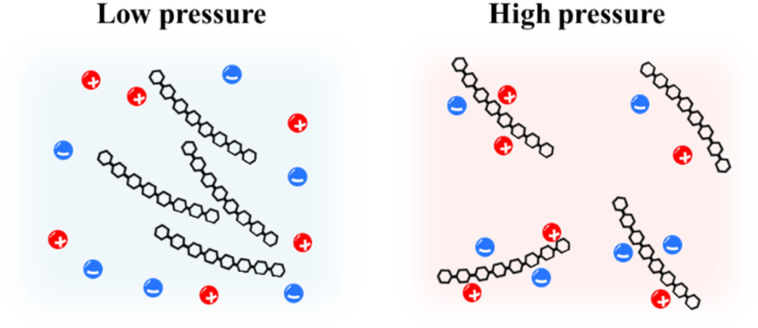
Schematic of the solution structures under relatively low and high pressures.

The molecular-level insights provided in Sub-sections (a)–(e) allowed us to propose a mechanism for the effect of pressure on cellulose solubility, as illustrated in Fig. 8. As the pressure increased, the interactions between the cellulose and the solvent, particularly the ionic liquid components, were significantly strengthened. This enhanced solvation of the individual cellulose chains by ions creates more robust solvent shells. These shells act as barriers, preventing cellulose chains from approaching each other and aggregating. Ultimately, this results in a more uniform and dispersed dissolution of cellulose under high-pressure conditions, which reduces unfavorable cellulose–cellulose interactions (aggregation).

This molecular mechanism is fully consistent with the thermodynamic result from Section 3.2.3, providing a clear rationale for why the dissolution free energy (Δ*G*_dis_) becomes more favorable under pressure and supporting our central conclusion of pressure-enhanced cellulose solubility.

#### 3.3.3 “Microscale” analysis of the cellulose structure in the [EMIm][OAc]/DMSO system

We analyzed the hydrogen bonds between the cellulose chains and solvent species, together with the dihedral angles of cellulose, to characterize the pressure-induced conformational changes and gain deeper molecular insights into pressure effects.

##### 3.3.3.1 Hydrogen bond network analysis

**Fig. 9:**
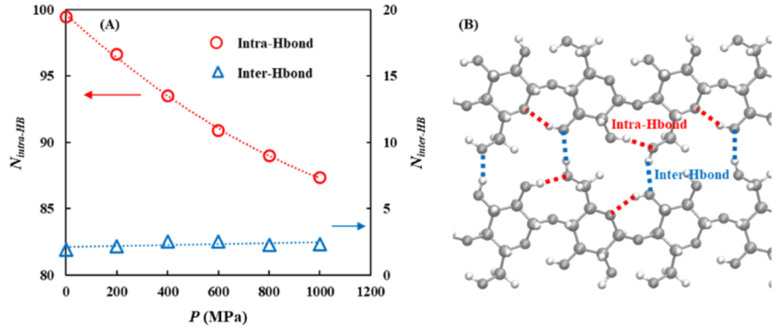
(A) Pressure dependence of the number of intra-hydrogen bonds (*N*_intra-HB_) between the cellulose and the number of inter-hydrogen bonds (*N*_inter-HB_) within the cellulose at *P* = 0.1, 200, 400, 600, 800, and 1000 MPa. (B) Schematic showing the structure of the intra-hydrogen bonds between the cellulose and inter-hydrogen bonds within the cellulose.

**Fig. 10:**
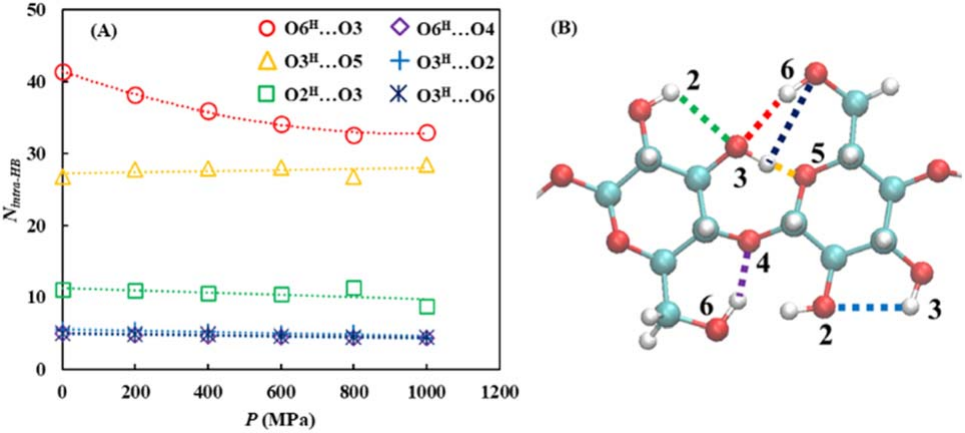
(A) Pressure dependence of the number of intra-hydrogen bonds (*N*_intra-HB_) within cellulose under *P* = 0.1, 200, 400, 600, 800, and 1000 MPa. (B) Schematic showing the structure of the intra-hydrogen bonds within the cellulose.

First, the pressure dependence of the hydrogen bond network was investigated by analyzing the intramolecular (within the cellulose) and intermolecular (between different molecules) hydrogen bonds. Although the number of intermolecular hydrogen bonds showed negligible pressure dependence, the number of intramolecular hydrogen bonds decreased by approximately 12% as the pressure increased to *P* = 1000 MPa [Fig. 9(A) and Table S24]. A more detailed analysis revealed that the O6^H^⋯O3 intramolecular hydrogen bond, which is generally recognized as the most dominant hydrogen-bonding motif within cellulose chains, showed the largest decrease with the increasing pressure [Fig. 10(A) and Table S25].

**Fig. 11:**
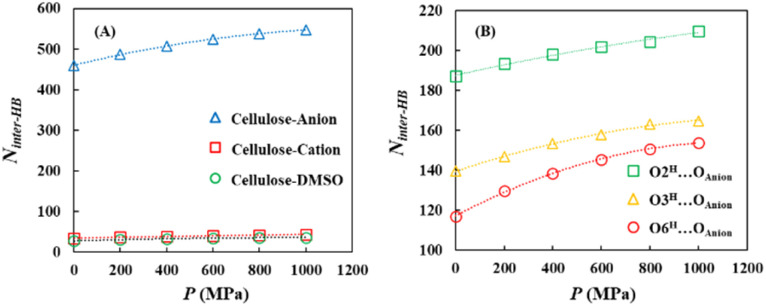
Pressure dependence of the number of inter-hydrogen bonds (A) between the cellulose and the solvent (anion, cation, and DMSO) and (B) between the cellulose and the anion under *P* = 0.1, 200, 400, 600, 800, and 1000 MPa.

In contrast, the number of intermolecular hydrogen bonds between cellulose and the solvent increased with increasing pressure, with the most pronounced increase observed for cellulose–anion interactions. These were not only the most abundant but also exhibited the strongest pressure dependence [Fig. 11(A) and Table S26]. Specifically, within the cellulose–anion hydrogen bonds, the O6^H^⋯O_anion_ hydrogen bond demonstrated the strongest pressure sensitivity [Fig. 11(B) and Table S27].

In summary, increasing pressure primarily reduced the number of O6^H^⋯O3 intramolecular hydrogen bonds while promoting the formation of O6^H^⋯O_anion_ intermolecular hydrogen bonds. This suggests that the C6 hydroxymethyl (CH_2_OH) group undergoes a pressure-induced transition from forming intramolecular hydrogen bonds within the cellulose chain to forming intermolecular hydrogen bonds with the anions. This behavior may be attributed either to a pressure-induced conformational change of the CH_2_OH group or to enhanced interactions between the cellulose and solvent species due to system compression.

##### 3.3.3.2 Pressure-induced conformational changes

**Fig. 12:**
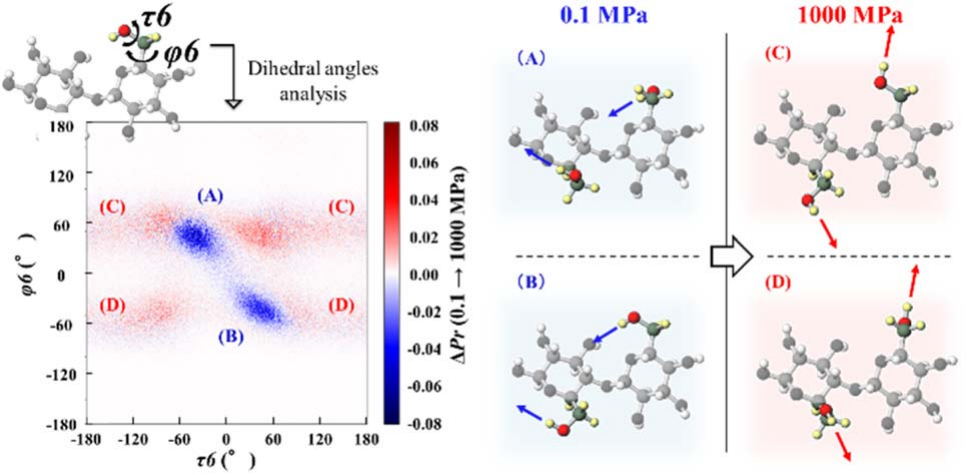
(Left) Two-dimensional probability difference map illustrating the variation in the joint distribution of the dihedral angles *φ*6 (O5–C5–C6–O6) and *τ*6 (C5–C6–O6–H6) between *P* = 0.1 and 1000 MPa. The blue and red regions indicate decreased and increased conformer populations, respectively. (Right) Representative cellulose conformations labeled (A)–(D), corresponding to the specific regions marked in the left panel. The arrows show the orientation vector of the C6 hydroxyl group.

Conformational analysis was conducted to clarify this point. Specifically, the dihedral angles related to the C6 position—*φ*6 (O5–C5–C6–O6) and *τ*6 (C5–C6–O6–H6)—were analyzed (Fig. S8). The left panel of Fig. 12 shows a two-dimensional probability difference map, which illustrates how the joint distribution of the two dihedral angles (*φ*6 and *τ*6) changed from *P* = 0.1 to 1000 MPa. The blue regions [(A) and (B)] indicate conformations that become less populated as pressure increased, whereas the red regions [(C) and (D)] indicate conformations that become more populated as pressure increased. The right panel depicts representative conformations associated with each region, where the arrows indicate the orientation vector of the hydroxyl group at the C6 position. As the pressure increases, the C6–OH vector, which initially points “inward” toward the cellulose backbone—an orientation favorable for forming intramolecular hydrogen bonds—gradually rotates “outward”. This outward orientation is favorable for establishing intermolecular hydrogen bonds with solvent molecules. Consequently, the population of the anti-conformer increases under high-pressure conditions, leading to a more solvent-exposed hydroxyl group configuration.

##### 3.3.3.3 Summary and proposed mechanism for pressure-enhanced solubility at “microscale”

**Fig. 13:**
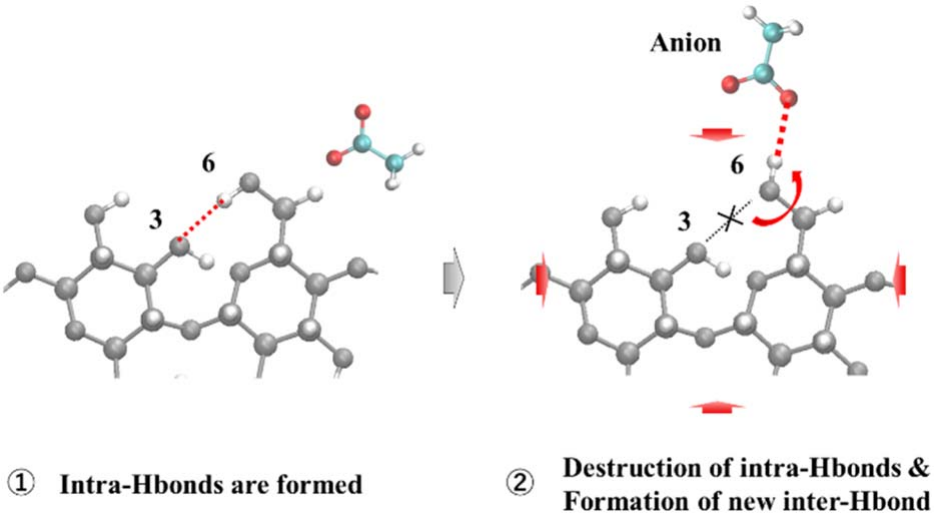
Mechanism of pressure-induced enhancement of cellulose solubility. Hydrogen bonds switch from cellulose intramolecular hydrogen bonds (intra-Hbonds) to intermolecular hydrogen bonds (inter-Hbonds) between the cellulose and the anions.

By integrating the results of the hydrogen bonding and conformational analyses of cellulose, the following microscopic molecular mechanism can be proposed to explain the pressure-enhanced solubility of cellulose (Fig. 13).

At ambient pressure, cellulose molecules form intramolecular O6^H^⋯O3 hydrogen bonds with a certain probability. However, under high pressure, the system equilibrium shifts toward states with smaller partial molar volumes in accordance with Le Chatelier's principle.

The conformational rotation of the hydroxymethyl (C6–OH) group, coupled with the penetration of [OAc]^−^ anions, leads to a more compact packing of the solvation shell, thereby reducing the total system volume (Δ*V* < 0). This volume-driven conformational change is analogous to pressure-induced protein denaturation, in which structural unfolding is favored to minimize the specific volume through enhanced hydration.

Crucially, this process is not driven by volume reduction alone but by a synergistic effect: the thermodynamic drive to reduce the volume brings the anion and cellulose into close contact, while the specific high hydrogen bond affinity of the anion energetically stabilizes the rotated conformation. As illustrated in Fig. 13, this leads to a concerted mechanism in which the disruption of the intramolecular bond and the formation of a stable intermolecular O6^H^⋯O_anion_ bond occur simultaneously.

Therefore, the enhanced solubility of cellulose observed under high-pressure conditions is not merely a passive consequence of system compression, as described in Section 3.3.2, but can also be attributed to an active, conformationally driven process in the cellulose molecule that reorganizes the hydrogen-bonding network to favor solvation.

## 4 Conclusion

In this study, AA MD simulations were used to predict the solubility of cellulose in a 60 wt% [EMIm][OAc]/DMSO solvent over a wide pressure range (*P* = 0.1–1000 MPa). Comprehensive analyses were conducted to elucidate the molecular mechanisms by which pressure influences cellulose solubility.

First, umbrella sampling simulations were performed to calculate the dissolution free energy (Δ*G*_dis_) and to predict the solubility of cellulose at higher pressures. The results revealed that Δ*G*_dis_ decreases monotonically as the pressure increases, which provides clear thermodynamic evidence that higher pressures enhance the solubility of cellulose in the [EMIm][OAc]/DMSO system.

The MD simulations of the dissolved cellulose system were analyzed in detail to elucidate the molecular origin of this behavior. The interaction energies, coordination numbers, and radial distribution functions showed that increasing the pressure strengthened cellulose–solvent interactions and weakened cellulose–cellulose interactions. Furthermore, analysis of the spatial distribution homogeneity demonstrated that the cellulose chains became more uniformly dispersed at higher pressures, forming a more homogeneous and well-solvated polymer solution. Together, these results indicate that high pressures reinforce ion–cellulose interactions, stabilize the solvation shells around each polymer chain, and simultaneously suppress cellulose–cellulose aggregation, thereby promoting dissolution under compression.

A deeper investigation into hydrogen bonding and conformational dynamics further revealed a more intricate active mechanism. Increasing the pressure-induced conformational transitions of the CH_2_OH groups, which disrupted the critical intramolecular hydrogen bond (O6^H^⋯O3) and facilitated the formation of new, favorable intermolecular hydrogen bonds between the cellulose and solvent anions.

Thus, pressure enhances cellulose solubility through passive densification and enhanced solvation, and active pressure-induced conformational remodeling of the hydrogen bond network thermodynamically favors dissolution.

From a practical perspective, improved solubility under pressure implies reduced solvent consumption, leading to potential economic and environmental benefits for cellulose processing. Overall, this study provides new fundamental insights into how pressure—a key thermodynamic parameter—governs solubility. The molecular-level understanding obtained in this study offers valuable guidance for designing next-generation biorefinery processes and sustainable material technologies. Specifically, the finding that cellulose–anion interactions exhibit the highest-pressure sensitivity suggests a clear strategy for solvent design: for high-pressure applications, ionic liquids containing anions with smaller steric bulk and higher hydrogen bond basicity, such as formate ([HCOO]^−^) or chloride ([Cl]^−^), can be selected to maximize the pressure-induced solvation enhancement.

## Author contributions

Kodai Kikuchi: conceptualization, methodology, investigation, data curation, writing–original draft, funding acquisition. Kazushi Fujimoto: methodology, supervision, writing-reviewing. Kazuyoshi Kaneko: methodology, supervision, writing-reviewing, funding acquisition. Akio Shimizu: conceptualization, funding acquisition. Tatsushi Matsuyama: methodology. Junichi Ida: methodology, writing-reviewing and editing, supervision, funding acquisition.

## Conflicts of interest

The authors declare that they have no known competing financial interests or personal relationships that may have influenced the work reported in this paper.

## Supplementary Material

RA-016-D5RA08753H-s001

## Data Availability

The datasets generated and/or analyzed during the current study are available from the corresponding author on reasonable request. Supplementary information (SI) is available. See DOI: https://doi.org/10.1039/d5ra08753h.
