# Supplementary material for: Prediction and elucidation of cellulose solubility in ionic liquids under high pressure using all-atom molecular dynamics simulations
Source: RSC Adv. 2026 Jan 13;16(3):2710–22. doi: 10.1039/d5ra08753h (PMC12797026; doi:10.1039/d5ra08753h)
Supplement: RA-016-D5RA08753H-s001 [file RA-016-D5RA08753H-s001.pdf]

Supplementary Information (SI) for RSC Advances.
This journal is © The Royal Society of Chemistry 2026

SUPPLEMENTARY INFORMATION

Prediction and Elucidation of Cellulose Solubility in Ionic Liquids under High Pressure using All-Atom Molecular Dynamics Simulations

Received 00th January 20xx,
Accepted 00th January 20xx

DOI: 10.1039/x0xx00000x

Kodai Kikuchi,[a*] Kazushi Fujimoto,[b] Kazuyoshi Kaneko,[c] Akio Shimizu,[a] Tatsushi Matsuyama [a] and Junichi Ida [a*]

[a.] *Environmental Engineering for Symbiosis, Graduate School of Science and Engineering, Soka University, 1-236 Tangi, Hachioji, Tokyo 192-8577, Japan.*
[b.] *Department of Chemistry and Materials Engineering, Faculty of Chemistry, Materials and Bioengineering, Kansai University, 3-3-35 Yamate-cho Suita, Osaka 564-8680, Japan.*
[c.] *Department of Science and Engineering for Sustainable Innovation, Faculty of Science and Engineering, Soka University, 1-236 Tangi, Hachioji, Tokyo 192-8577, Japan.*
* Corresponding author
Email: e24D5802@soka-u.jp (K. Kikuchi)
Email: ida@soka.ac.jp (J. Ida)

Please do not adjust margins

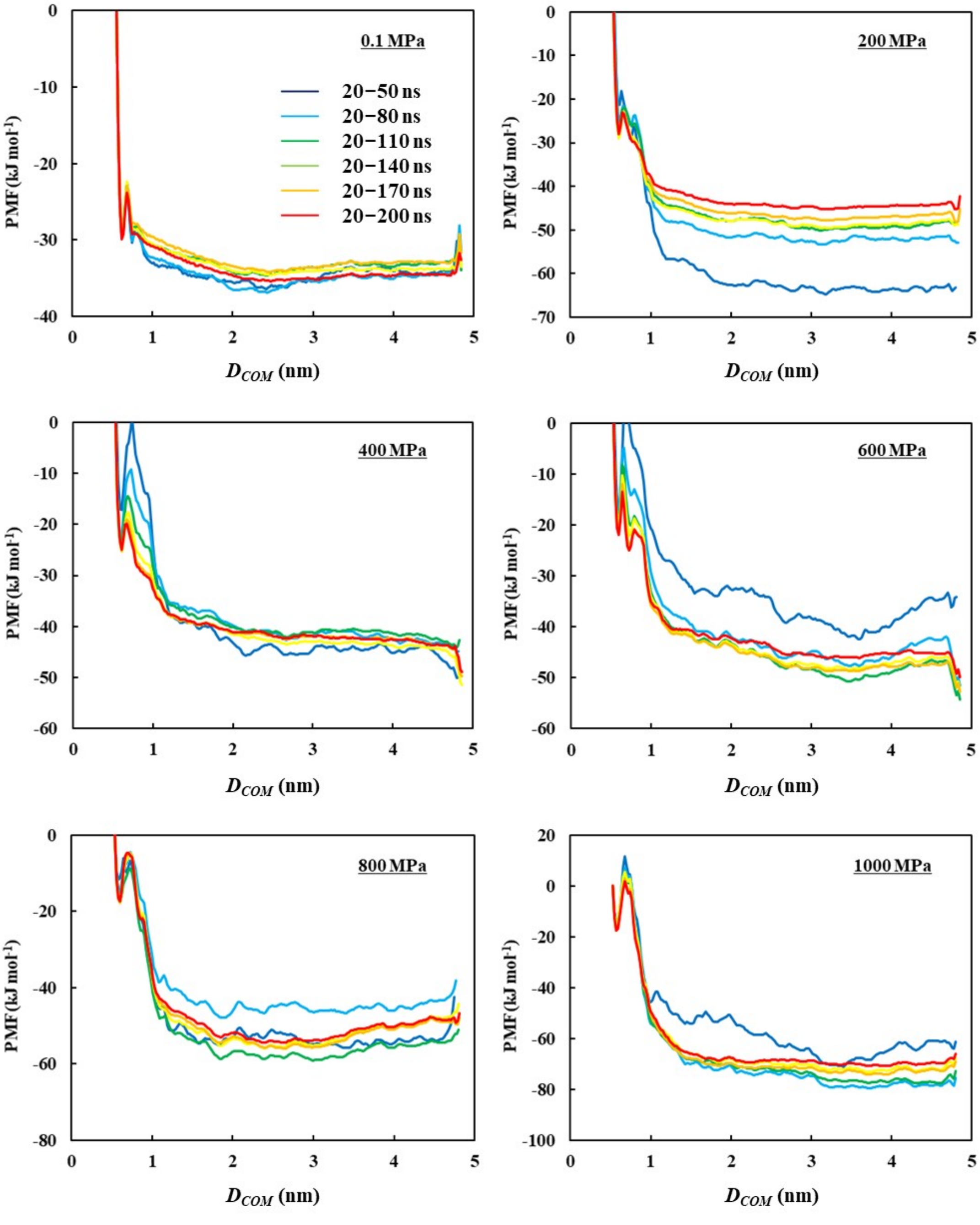

Fig. S1. Potential of Mean Force (PMF) as a function of the center-of-mass distance (D_{COM}) between the crystalline cellulose and a single cellulose chain estimated for each WHAM time range (20–50, 20–80, 20–110, 20–140, 20–170, and 20–200 ns) under P = 0.1, 200, 400, 600, 800, and 1000 MPa.

 This journal is © The Royal Society of Chemistry 20xx

Please do not adjust margins

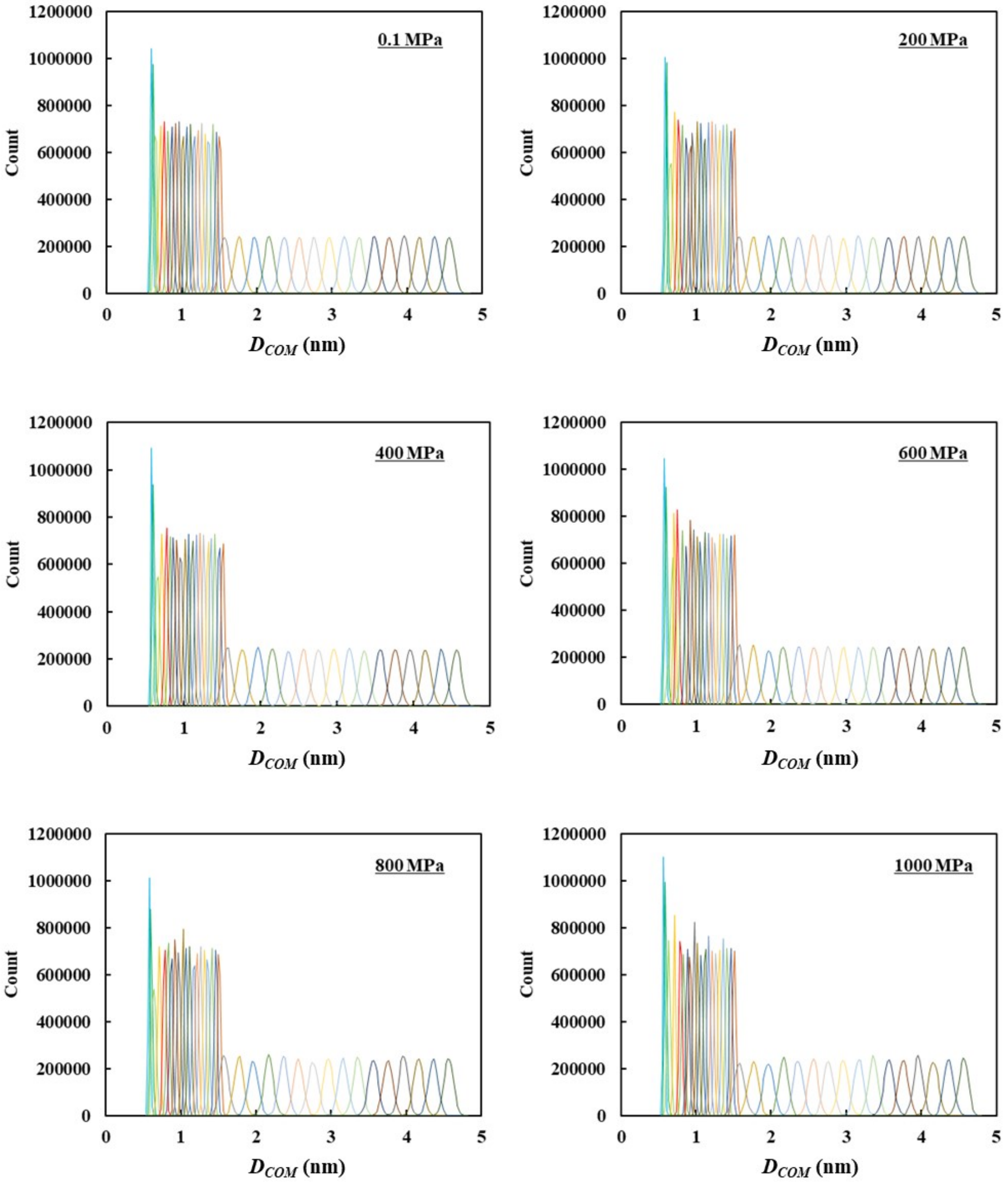

Fig. S2. Umbrella sampling histograms for each window (WHAM range 20–200 ns) under *P* = 0.1, 200, 400, 600, 800, and 1000 MPa.

Please do not adjust margins

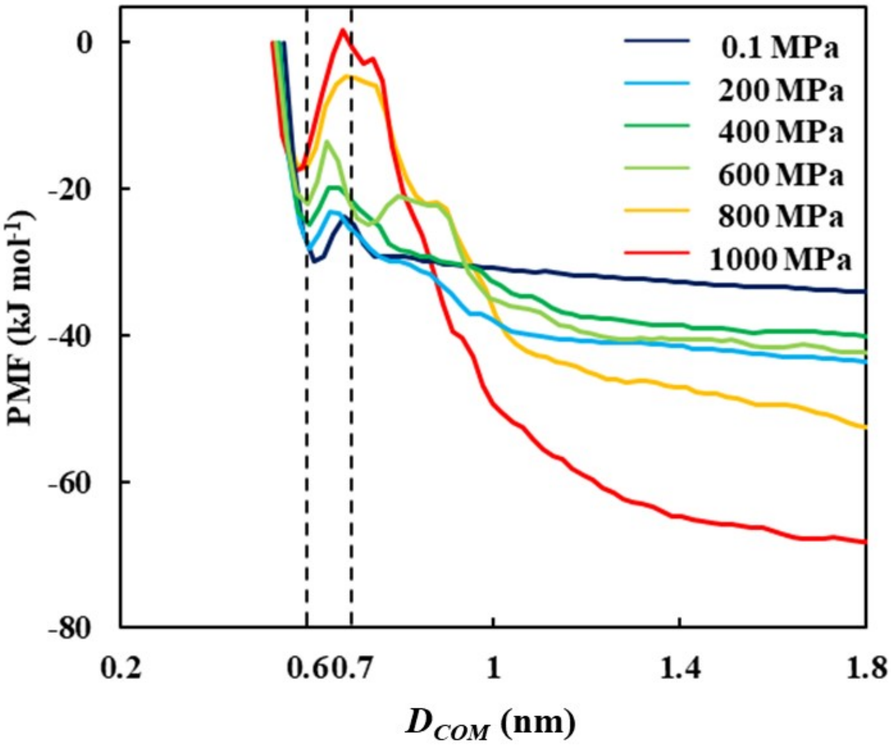

Fig. S3. Pressure dependence of the magnified PMF (0.2–1.8 nm) under *P* = 0.1, 200, 400, 600, 800, and 1000 MPa.

This journal is © The Royal Society of Chemistry 20xx

Please do not adjust margins

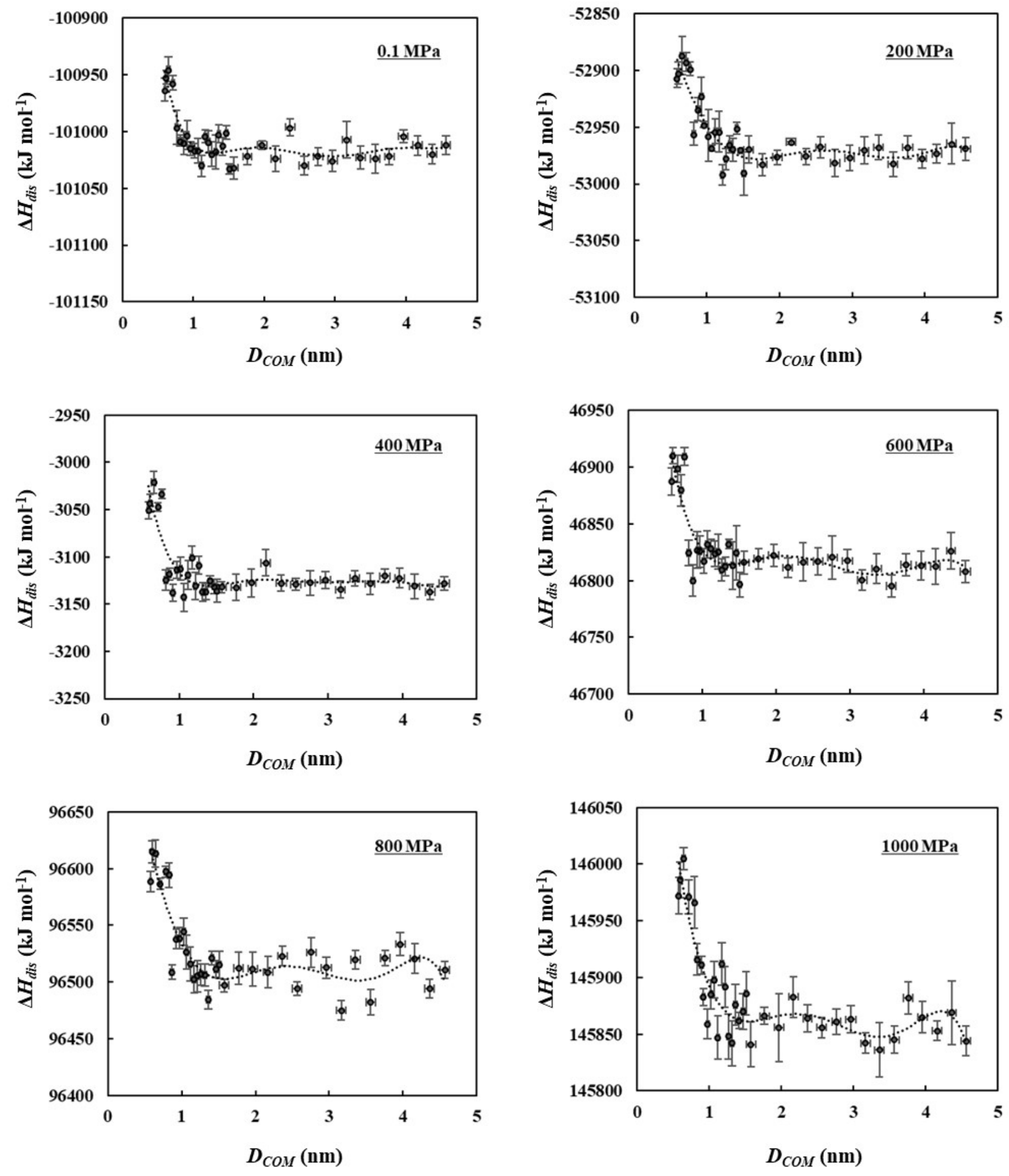

Fig. S4. Dissolution enthalpy profile under P = 0.1, 200, 400, 600, 800, and 1000 MPa. Error bars represent the estimated error.

Please do not adjust margins

Please do not adjust margins

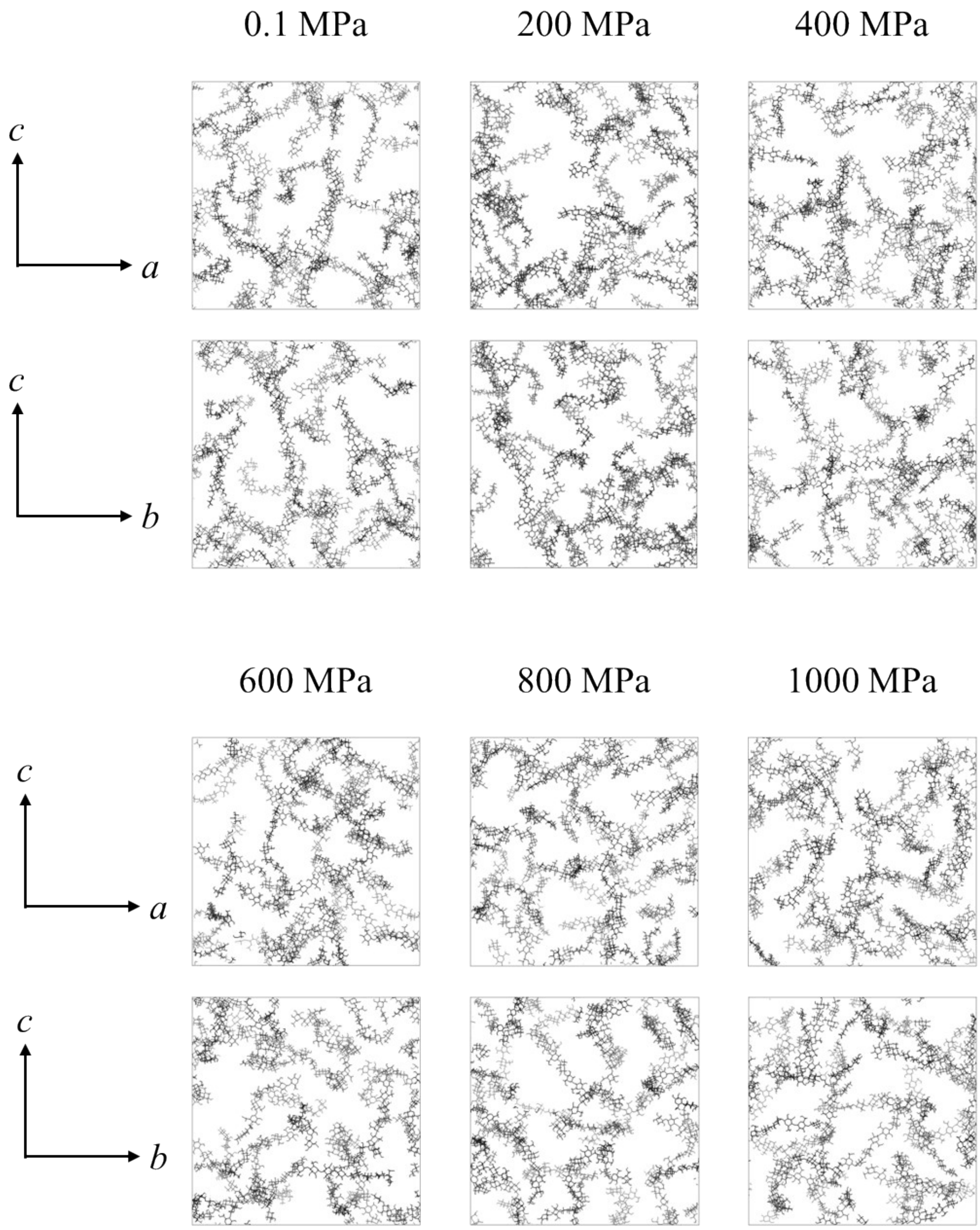

Fig. S5. Snapshots of the cellulose structures dissolved in the ionic liquid/DMSO solvent after a 100 ns *NPT* production run under *P* = 0.1, 200, 400, 600, 800, and 1000 MPa (solvents are hidden).

This journal is © The Royal Society of Chemistry 20xx

Please do not adjust margins

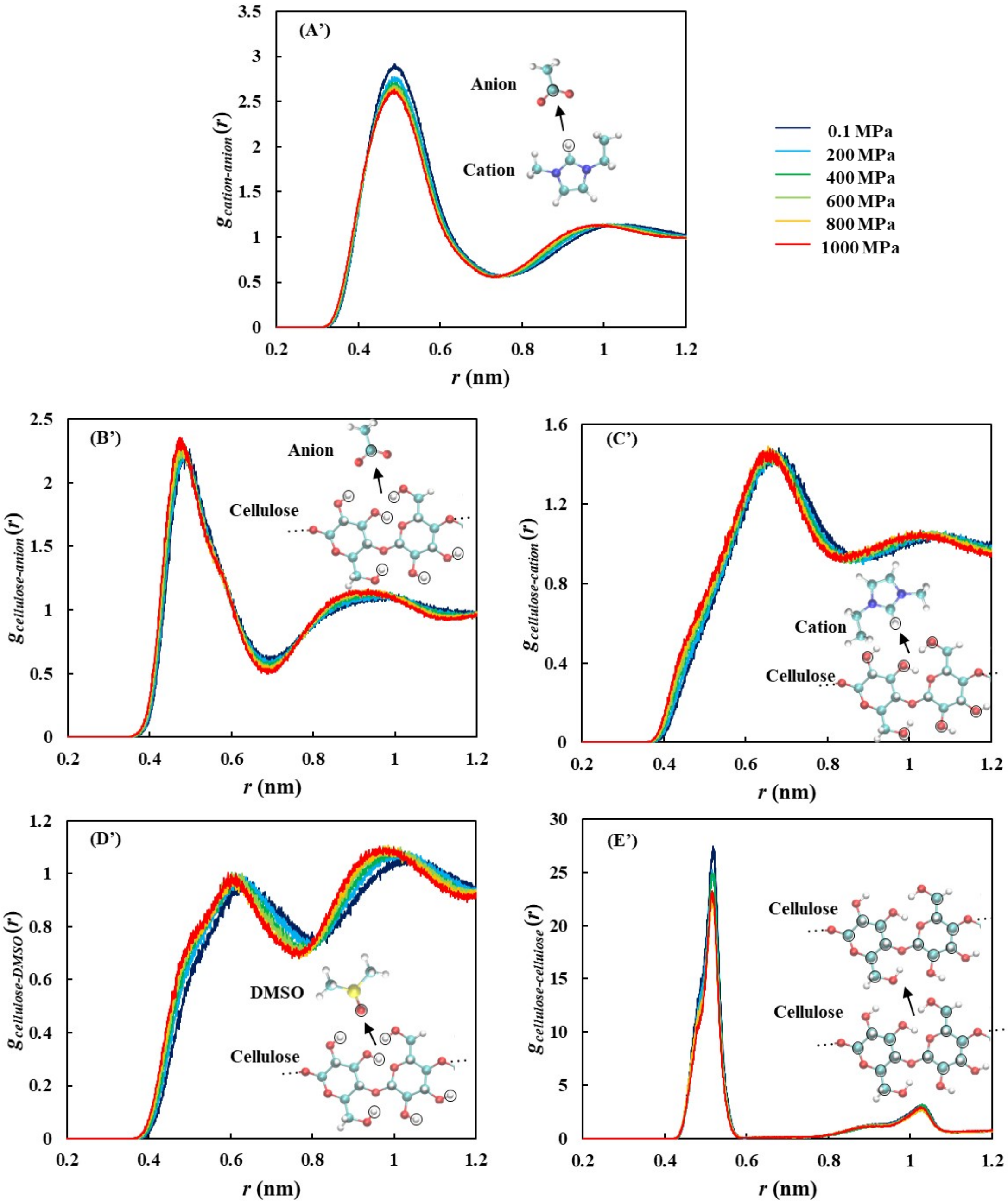

Fig. S6. RDF $g(r)$ for (A') cation–anion, (B') cellulose–anion, (C') cellulose–cation, (D') cellulose–DMSO, and (E') cellulose–cellulose under P = 0.1, 200, 400, 600, 800, and 1000 MPa. The reference and target atoms for the RDF are defined in Section 2.3.2.

Please do not adjust margins

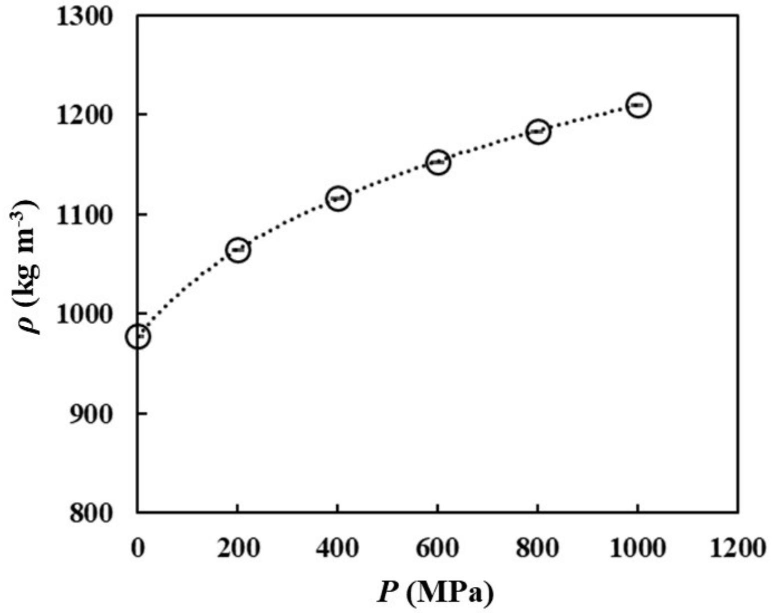

Fig. S7. Pressure dependence of the density (ρ) of the cellulose solution (cellulose/[EMIm][OAc]/DMSO) under P = 0.1, 200, 400, 600, 800, and 1000 MPa.

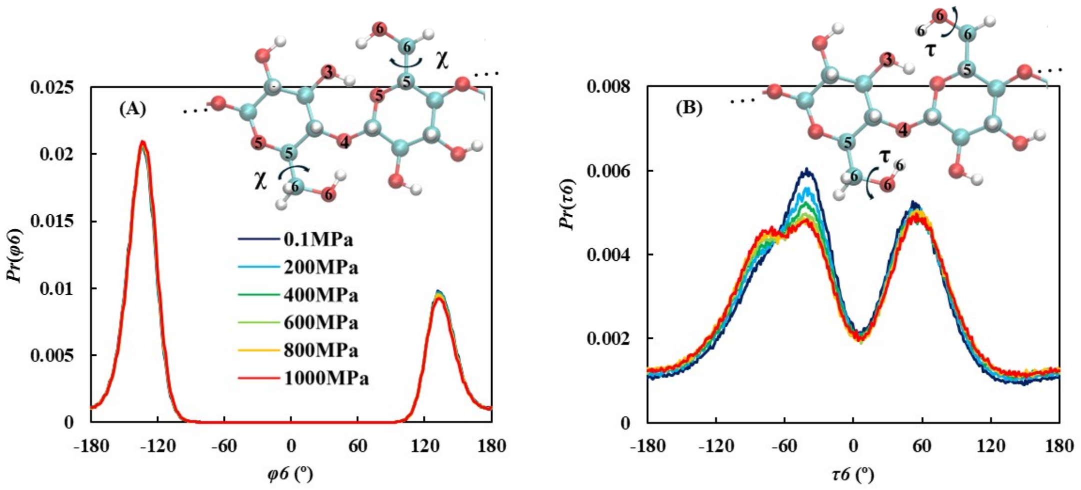

Fig. S8. Pressure dependence of the dihedral angle distributions of (A) $\varphi6$(O5-C5-C6-O6) and (B) $\tau6$(C5-C6-O6-H6) under P = 0.1, 200, 400, 600, 800, and 1000 MPa.

This journal is © The Royal Society of Chemistry 20xx

Please do not adjust margins

Please do not adjust margins

Table S1. Calculation conditions for the umbrella sampling simulations. The center-of-mass distance (D_{COM}) and corresponding spring constant (k_{umb}) for each sampling window are listed. The umbrella potential was applied harmonically along the reaction coordinate defined by D_{COM}.

Windows No.	0	1	2	3	4	5	6	7	8
D_{COM} (nm)	0.6	0.65	0.7	0.75	0.8	0.85	0.9	0.95	1
k_{umb} (kJ mol^{-1} nm^2)	10,000	10,000	10,000	10,000	10,000	10,000	10,000	10,000	10,000
Windows No.	9	10	11	12	13	14	15	16	17
D_{COM} (nm)	1.05	1.1	1.15	1.2	1.25	1.3	1.35	1.4	1.45
k_{umb} (kJ mol^{-1} nm^2)	10000	1000	1000	1000	1000	1000	1000	1000	1000
Windows No.	18	19	20	21	22	23	24	25	26
D_{COM} (nm)	1.5	1.55	1.6	1.8	2	2.2	2.4	2.6	2.8
k_{umb} (kJ mol^{-1} nm^2)	1000	1000	1000	1000	1000	1000	1000	1000	1000
Windows No.	27	28	29	30	31	32	33	34	35
D_{COM} (nm)	3	3.2	3.4	3.6	3.8	4	4.2	4.4	4.6
k_{umb} (kJ mo^{-1} nm^2)	1000	1000	1000	1000	1000	1000	1000	1000	1000

Table S2. Solution densities (ρ) of [EMIm][OAc] obtained from the MD simulations and experiments [50, 51] at each temperature (T) and pressure (P) and their relative errors.

T (K)	P (MPa)	ρ (kg m^{-3})		Relative error (%)
		MD	Exp. [Ref.]	
293	0.1	1104.28	1101.7 [50]	–0.0290
323	0.1	1082.05	1083.7 [50]	–0.0526
373	0.1	1045.54	1054.2 [50]	–0.1081
323	50	1095.22	1100.3 [51]	–0.4617
323	100	1106.18	1116.6 [51]	–0.9332

Table S3. Solution densities (ρ) of DMSO obtained from the MD simulations and experiments [52] at each temperature (T) and pressure (P) and their relative errors.

T (K)	P (MPa)	ρ (kg m^{-3})		Relative error (%)
		MD	Exp. [Ref.]	
293	0.1	1097.66	1098.5 [52]	–0.0765
333	0.1	1058.27	1061.0 [52]	–0.2573
373	0.1	1018.63	1019.9 [52]	–0.1245
333	15	1065.76	1069.9 [52]	–0.3870
333	35	1075.12	1081.7 [52]	–0.6083

Please do not adjust margins

Please do not adjust margins

Table S4. Solution densities (ρ) of 60 wt% [EMIm][OAc]/DMSO obtained from the MD simulations and experiments [50] at each temperature (T) and their relative errors.

T (K)	P (MPa)	ρ (kg m^{-3})		Relative error (%)
		MD	Exp. [Ref.]	
293	0.1	1104.28	1107.4 [50]	–0.2817
323	0.1	1082.05	1085.1 [50]	–0.2811
373	0.1	1045.54	1048.5 [50]	–0.2823

Table S5. Free energy components, including the crystal (G_{xtal}), activated ($G^‡$), solvated (G_{solv}), activation (ΔG_{act}), and dissolution (ΔG_{dis}) free energies, calculated from the WHAM analysis over different simulation time ranges under P = 0.1 MPa.

WHAM time range (ns)	20–50	50–80	80–110	110–140	140–170	170–200	Average	SD
G_{xtal} (kJ mol^{-1})	–28.74	–29.14	–27.74	–26.52	–29.74	–34.15	–29.34	2.62
$G^‡$ (kJ mol^{-1})	–23.14	–20.22	–20.61	–20.63	–22.63	–29.32	–22.76	3.43
G_{solv} (kJ mol^{-1})	–34.11	–34.36	–28.84	–34.34	–29.66	–43.70	–34.17	5.28
ΔG_{act} (kJ mol^{-1})	5.60	8.93	7.13	5.89	7.11	4.82	6.58	1.46
ΔG_{dis} (kJ mol^{-1})	–5.37	–5.21	–1.10	–7.82	0.09	–9.55	–4.83	3.73

Table S6. Free energy components, including the crystal (G_{xtal}), activated ($G^‡$), solvated (G_{solv}), activation (ΔG_{act}), and dissolution (ΔG_{dis}) free energies, calculated from the WHAM analysis over different simulation time ranges under P = 200 MPa.

WHAM time range (ns)	20–50	50–80	80–110	110–140	140–170	170–200	Average	SD
G_{xtal} (kJ mol^{-1})	–23.62	–24.90	–28.90	–27.88	–20.95	–25.33	–25.26	2.88
$G^‡$ (kJ mol^{-1})	–18.09	–16.34	–19.72	–21.34	–17.45	–19.42	–18.73	1.79
G_{solv} (kJ mol^{-1})	–63.70	–37.19	–41.77	–41.47	–37.40	–27.69	–41.54	11.99
ΔG_{act} (kJ mol^{-1})	5.54	8.55	9.19	6.54	3.50	5.91	6.54	2.08
ΔG_{dis} (kJ mol^{-1})	–40.08	–12.29	–12.87	–13.59	–16.45	–2.36	–16.27	12.61

This journal is © The Royal Society of Chemistry 20xx

Please do not adjust margins

Please do not adjust margins

Table S7. Free energy components, including the crystal (G_{xtal}), activated ($G^{‡}$), solvated (G_{solv}), activation (ΔG_{act}), and dissolution (ΔG_{dis}) free energies, calculated from the WHAM analysis over different simulation time ranges under P = 400 MPa.

WHAM time range (ns)	20–50	50–80	80–110	110–140	140–170	170–200	Average	SD
G_{xtal} (kJ mol^{-1})	–17.13	–24.23	–24.98	–22.86	–22.26	–29.98	–23.57	4.18
$G^{‡}$ (kJ mol^{-1})	0.87	–13.24	–19.54	–17.33	–15.52	–21.66	–14.40	8.04
G_{solv} (kJ mol^{-1})	–44.24	–34.10	–38.03	–44.79	–33.26	–43.81	–39.70	5.27
ΔG_{act} (kJ mol^{-1})	18.00	10.99	5.43	5.52	6.75	8.32	9.17	4.80
ΔG_{dis} (kJ mol^{-1})	–27.11	–9.87	–13.06	–21.93	–11.00	–13.83	–16.13	6.85

Table S8. Free energy components, including the crystal (G_{xtal}), activated ($G^{‡}$), solvated (G_{solv}), activation (ΔG_{act}), and dissolution (ΔG_{dis}) free energies, calculated from the WHAM analysis over different simulation time ranges under P = 600 MPa.

WHAM time range (ns)	20–50	50–80	80–110	110–140	140–170	170–200	Average	SD
G_{xtal} (kJ mol^{-1})	–17.35	–16.63	–28.18	–22.37	–28.70	–26.39	–23.27	5.35
$G^{‡}$ (kJ mol^{-1})	8.17	–8.03	–12.95	–13.68	–13.45	–16.52	–9.41	9.04
G_{solv} (kJ mol^{-1})	–40.45	–47.33	–56.56	–41.03	–45.56	–28.55	–43.25	9.25
ΔG_{act} (kJ mol^{-1})	25.52	8.60	15.23	8.69	15.25	9.87	13.86	6.48
ΔG_{dis} (kJ mol^{-1})	–23.10	–30.70	–28.38	–18.66	–16.86	–2.16	–19.98	10.24

Table S9. Free energy components, including the crystal (G_{xtal}), activated ($G^{‡}$), solvated (G_{solv}), activation (ΔG_{act}), and dissolution (ΔG_{dis}) free energies, calculated from the WHAM analysis over different simulation time ranges under P = 800 MPa.

WHAM time range (ns)	20–50	50–80	80–110	110–140	140–170	170–200	Average	SD
G_{xtal} (kJ mol^{-1})	–11.63	–18.49	–21.56	–16.19	–14.07	–12.09	–15.67	3.86
$G^{‡}$ (kJ mol^{-1})	–6.09	–2.63	–15.02	11.77	6.82	3.78	–0.23	9.68
G_{solv} (kJ mol^{-1})	–54.77	–37.23	–77.62	–35.19	–51.14	–42.66	–49.77	15.64
ΔG_{act} (kJ mol^{-1})	5.55	15.86	6.54	27.95	20.89	15.87	15.44	8.53
ΔG_{dis} (kJ mol^{-1})	–43.14	–18.74	–56.06	–19.00	–37.07	–30.57	–34.10	14.48

Table S10. Free energy components, including the crystal (G_{xtal}), activated ($G^{‡}$), solvated (G_{solv}), activation (ΔG_{act}), and dissolution (ΔG_{dis}) free energies, calculated from the WHAM analysis over different simulation time ranges under P = 1000 MPa.

WHAM time range (ns)	20–50	50–80	80–110	110–140	140–170	170–200	Average	SD
G_{xtal} (kJ mol^{-1})	–14.22	–13.45	–13.28	–12.78	–18.54	–13.13	–14.23	2.17
$G^{‡}$ (kJ mol^{-1})	11.43	6.05	8.51	12.88	–1.07	4.83	7.10	5.04
G_{solv} (kJ mol^{-1})	–68.09	–86.17	–66.55	–57.26	–69.32	–49.86	–66.21	12.32
ΔG_{act} (kJ mol^{-1})	25.65	19.50	21.79	25.66	17.48	17.96	21.34	3.67
ΔG_{dis} (kJ mol^{-1})	–53.87	–72.72	–53.27	–44.48	–50.78	–36.73	–51.97	12.05

This journal is © The Royal Society of Chemistry 20xx

Please do not adjust margins

Please do not adjust margins

Table S11. Pressure dependence of the dissolution enthalpy (ΔH_{dis}). The average and error estimates for the crystal enthalpy (H_{xtal}) and solvation enthalpy (H_{solv}) are shown under P = 0.1, 200, 400, 600, 800, and 1000 MPa.

P (MPa)	H_{xtal} (kJ mol^{-1})		H_{solv} (kJ mol^{-1})		ΔH_{dis} (kJ mol^{-1})	
	Average	Err. Est.	Average	Err. Est.	Average	Err. Est.
0.1	–100,963	10	–101,019	8	–56	18
200	–52,907	8	–52,973	6	–66	14
400	–3051	9	–3128	6	–77	15
600	46,888	12	46,813	9	–75	21
800	96,589	11	96,508	20	–81	31
1000	145,972	16	145,857	14	–115	30

Table S12. Pressure dependence of the dissolution free energy (ΔG_{dis}), dissolution enthalpy (ΔH_{dis}), and dissolution entropic term ($-T\Delta S_{dis}$). The average and standard deviation of each property are shown under P = 0.1, 200, 400, 600, 800, and 1000 MPa.

P (MPa)	ΔG_{dis} (kJ mol^{-1})		ΔH_{dis} (kJ mol^{-1})		$-T\Delta S_{dis}$ (kJ mol^{-1})	
	Average	SD	Average	Err. Est.	Average	SD
0.1	–4.83	3.73	–56	18	51	22
200	–16.27	12.61	–66	14	50	27
400	–16.13	6.85	–77	15	61	21
600	–19.98	10.24	–75	21	55	31
800	–34.10	14.48	–81	31	47	45
1000	–51.97	12.05	–115	30	63	42

Table S13. Pressure dependence of Coulomb interaction energy ($U_{Coulomb}$), van der Waals interaction energy (U_{vdW}), and interaction energy ($U_{nonbonded}$) between cation and anion under P = 0.1, 200, 400, 600, 800, and 1000 MPa.

P (MPa)	$U_{Coulomb}$ (kJ mol^{-1})		U_{vdW} (kJ mol^{-1})		$U_{nonbonded}$ (kJ mol^{-1})	
	Average	Err. Est.	Average	Err. Est.	Average	Err. Est.
0.1	–99,363.8	14	–30,572.9	7.3	–129,936.7	21.3
200	–102,862	27	–31,571.6	14	–134,433.6	41
400	–105,667	27	–31,760.4	13	–137,427.4	40
600	–108,032	21	–31,617.6	12	–139,649.6	33
800	–110,052	73	–31,277.2	28	–141,329.2	101
1000	–111,971	46	–30,834	12	–142,805	58

Table S14. Pressure dependence of the coordination number (CN) of the anions around the cation and their integration ranges under P = 0.1, 200, 400, 600, 800, and 1000 MPa.

P (MPa)	Integration range (nm)	CN
0.1	0–0.751	4.057
200	0–0.736	4.135
400	0–0.744	4.313
600	0–0.737	4.335
800	0–0.738	4.407
1000	0–0.736	4.447

This journal is © The Royal Society of Chemistry 20xx

Please do not adjust margins

Please do not adjust margins

Table S15. Pressure dependence of the Coulomb interaction energy ($U_{Coulomb}$), van der Waals interaction energy (U_{vdW}), and interaction energy ($U_{nonbonded}$) between the cellulose and anions under P = 0.1, 200, 400, 600, 800, and 1000 MPa.

P (MPa)	$U_{Coulomb}$ (kJ mol^{-1})		U_{vdW} (kJ mol^{-1})		$U_{nonbonded}$ (kJ mol^{-1})	
	Average	Err. Est.	Average	Err. Est.	Average	Err. Est.
0.1	–26,632	19	–1780	6	–28,411	25
200	–28,397	28	–1851	6	–30,248	34
400	–29,734	68	–1837	11	–31,571	79
600	–30,837	57	–1735	4	–32,572	61
800	–31,757	49	–1645	24	–33,402	73
1000	–32,518	38	–1550	9	–34,069	47

Table S16. Pressure dependence of the coordination number (CN) of the cellulose around the anions and their integration ranges under P = 0.1, 200, 400, 600, 800, and 1000 MPa.

P (MPa)	Integration range (nm)	CN
0.1	0–0.685	2.454
200	0–0.691	2.694
400	0–0.698	2.888
600	0–0.680	2.853
800	0–0.696	3.050
1000	0–0.690	3.083

Table S17. Pressure dependence of Coulomb interaction energy ($U_{Coulomb}$), van der Waals interaction energy (U_{vdW}), and interaction energy ($U_{nonbonded}$) between the cellulose and cations under P = 0.1, 200, 400, 600, 800, and 1000 MPa.

P (MPa)	$U_{Coulomb}$ (kJ mol^{-1})		U_{vdW} (kJ mol^{-1})		$U_{nonbonded}$ (kJ mol^{-1})	
	Average	Err. Est.	Average	Err. Est.	Average	Err. Est.
0.1	–2218	3	–8027	9	–10,246	12
200	–2558	3	–8660	17	–11,219	20
400	–2818	16	–9050	24	–11,868	40
600	–3005	5	–9176	22	–12,182	27
800	–3182	12	–9316	27	–12,499	39
1000	–3346	14	–9344	16	–12,689	30

Table S18. Pressure dependence of the coordination number (CN) of the cellulose around the cations and their integration ranges under P = 0.1, 200, 400, 600, 800, and 1000 MPa.

P (MPa)	Integration range (nm)	CN
0.1	0–0.866	4.827
200	0–0.858	5.065
400	0–0.857	5.322
600	0–0.848	5.309
800	0–0.841	5.346
1000	0–0.844	5.520

Please do not adjust margins

Please do not adjust margins

Table S19. Pressure dependence of the Coulomb interaction energy ($U_{Coulomb}$), van der Waals interaction energy (U_{vdW}), and interaction energy ($U_{nonbonded}$) between the cellulose and DMSO under P = 0.1, 200, 400, 600, 800, and 1000 MPa.

P (MPa)	$U_{Coulomb}$ (kJ mol^{-1})		U_{vdW} (kJ mol^{-1})		$U_{nonbonded}$ (kJ mol^{-1})	
	Average	Err. Est.	Average	Err. Est.	Average	Err. Est.
0.1	−1970.55	6.3	−5690.46	12	−7661.01	18.3
200	−2276.55	4.3	−6277.22	18	−8553.77	22.3
400	−2436.56	20	−6465.25	49	−8901.81	69
600	−2569.06	14	−6650.77	32	−9219.83	46
800	−2655.78	31	−6710.04	43	−9365.82	74
1000	−2774.38	23	−6748.65	26	−9523.03	49

Table S20. Pressure dependence of the coordination number (CN) of the cellulose around the DMSO and their integration ranges under P = 0.1, 200, 400, 600, 800, and 1000 MPa.

P (MPa)	Integration range (nm)	CN
0.1	0–0.800	3.903
200	0–0.783	4.008
400	0–0.791	4.280
600	0–0.771	4.095
800	0–0.772	4.202
1000	0–0.768	4.216

Table S21. Pressure dependence of the coordination number (CN) of the cellulose around the DMSO and their integration ranges under P = 0.1, 200, 400, 600, 800, and 1000 MPa.

P (MPa)	Integration range (nm)	CN
0.1	0–0.600	1.758
200	0–0.600	1.761
400	0–0.600	1.761
600	0–0.600	1.762
800	0–0.600	1.762
1000	0–0.600	1.762

Table S22. Pressure dependence of the Coulomb interaction energy ($U_{Coulomb}$), van der Waals interaction energy (U_{vdW}), and interaction energy ($U_{nonbonded}$) between the cellulose and cellulose under P = 0.1, 200, 400, 600, 800, and 1000 MPa.

P (MPa)	$U_{Coulomb}$ (kJ mol^{-1})		U_{vdW} (kJ mol^{-1})		$U_{nonbonded}$ (kJ mol^{-1})	
	Average	Err. Est.	Average	Err. Est.	Average	Err. Est.
0.1	−105,152	6	−2993	3	−108,145	9
200	−104,433	13	−2957	12	−107,390	25
400	−103,972	14	−2916	9	−106,888	23
600	−103,632	28	−2874	12	−106,506	40
800	−103,403	29	−2816	10	−106,219	39
1000	−103,160	20	−2816	8	−105,976	28

This journal is © The Royal Society of Chemistry 20xx

Please do not adjust margins

Please do not adjust margins

Table S23. Pressure dependence of the density (ρ) of the cellulose solution under P = 0.1, 200, 400, 600, 800, and 1000 MPa.

P (MPa)	ρ (kg m^{-3})	
	Average	Err. Est.
0.1	978.03	0.01
200	1065.03	0.01
400	1115.83	0.02
600	1153.48	0.01
800	1183.97	0.01
1000	1209.79	0.01

Table S24. Pressure dependence of the number of intermolecular hydrogen bonds ($N_{inter\text{-}HB}$) and intramolecular hydrogen bonds ($N_{intra\text{-}HB}$) of cellulose molecules dissolved in the cellulose solution under P = 0.1, 100, 200, 400, 600, 800, and 1000 MPa.

P (MPa)	$N_{inter\text{-}HB}$	$N_{intra\text{-}HB}$
0.1	1.90	99.49
200	2.19	96.67
400	2.53	93.50
600	2.54	90.89
800	2.26	89.00
1000	2.32	87.34

Table S25. Pressure dependence of the number of each type of intra-cellulose hydrogen bonds under P = 0.1, 200, 400, 600, 800, and 1000 MPa.

P (MPa)	$N_{intra\text{-}HB}$				
	O6^H...O3	O3^H...O5	O2^H...O3	O6^H...O4	O3^H...O2
0.1	41.41	26.88	11.06	5.08	5.54
200	38.20	27.84	11.00	4.84	5.52
400	35.99	27.92	10.56	4.67	5.26
600	34.12	28.12	10.43	4.50	5.03
800	32.57	26.84	11.33	4.40	5.22
1000	32.99	28.51	8.75	4.44	4.44

Table S26. Pressure dependence of the number of hydrogen bonds ($N_{inter\text{-}HB}$) between the cellulose and solvents (anion, cation, and DMSO) under P = 0.1, 200, 400, 600, 800, and 1000 MPa.

P (MPa)	$N_{inter\text{-}HB}$ between cellulose and solvents		
	Anion	Cation	DMSO
0.1	460.13	33.74	27.72
200	487.53	37.01	31.62
400	508.34	39.39	33.54
600	524.27	40.90	35.05
800	538.20	41.97	35.55
1000	548.19	43.08	36.62

This journal is © The Royal Society of Chemistry 20xx

Please do not adjust margins

Please do not adjust margins

Table S27. Pressure dependence of the number of each type of hydrogen bond between the cellulose and anions at P = 0.1, 200, 400, 600, 800, and 1000 MPa.

P (MPa)	$N_{inter\text{-}HB}$ between cellulose and anion			
	Total	$O2^H...O_{anion}$	$O3^H...O_{anion}$	$O6^H...O_{anion}$
0.1	460.13	187.20	139.70	116.89
200	487.53	193.57	146.96	129.75
400	508.34	198.10	153.53	138.53
600	524.27	202.00	157.82	145.53
800	538.20	204.40	163.34	150.79
1000	548.19	209.84	164.92	153.98

 This journal is © The Royal Society of Chemistry 20xx

Please do not adjust margins
